# Can people detect errors in shadows and reflections?

**DOI:** 10.3758/s13414-019-01773-w

**Published:** 2019-06-28

**Authors:** Sophie J. Nightingale, Kimberley A. Wade, Hany Farid, Derrick G. Watson

**Affiliations:** 1 Department of Psychology, University of Warwick, Coventry, UK; 2 Department of Computer Science, Dartmouth College, Dartmouth, NH USA

**Keywords:** Image manipulation, Digital image forensics, Visual processing, Human perception

## Abstract

The increasing sophistication of photo-editing software means that even amateurs can create compelling doctored images. Yet recent research suggests that people’s ability to detect image manipulations is limited. Given the prevalence of manipulated images in the media, on social networking sites, and in other domains, the implications of mistaking a fake image as real, or vice versa, can be serious. In seven experiments, we tested whether people can make use of errors in shadows and reflections to determine whether or not an image has been manipulated. Our results revealed that people’s ability to identify authentic and manipulated scenes based on shadow and reflection information increased with the size of the manipulation, but overall, detection rates remained poor. Consistent with theories of incomplete visual representation, one possible reason for these findings could be that people rarely encode the details of scenes that provide useful cues as to the authenticity of images. Overall, our findings indicate that people do not readily make use of shadow and reflection cues to help determine the authenticity of images—yet it remains possible that people could make use of these cues, but they are simply unaware of how to do so.

On May 23, 2016, Dinesh and Tarakeshwari Rathod were hailed as the first Indian couple to conquer Mount Everest (Boone, 2016). Yet the couple’s celebrations were cut short when fellow mountaineers charged that the couple never made it to the summit and that the photos they provided as evidence of their success were forgeries. Of particular interest was the date and time stamp on the photos—6.25 a.m. on May 23, 2016. Crucially, the shadows in the image suggested the photo was taken closer to noon than to 6.25 a.m. (Boone, 2016). Following an investigation, the Nepalese government concluded that the couple had indeed faked their summit photos and subsequently banned them from mountaineering in Nepal for 10 years (Safi, 2016). The Rathod’s story highlights how shadow information offers a useful means to detect photo forgeries. In the present study, we examine whether people can use inconsistencies between shadows, and similarly, inconsistencies between reflections, within a single scene to determine if an image has been manipulated.

The growing sophistication of photo-editing software means nearly anyone can make a fairly convincing forgery. For instance, the phone app Facetune^®^ allows users to reshape noses, whiten teeth, remove blemishes, perfect skin, and even add a smile (King, 2015). In coming years, algorithms might be used to invent or fabricate entire scenes (Quach, 2017). Yet a growing body of research suggests that people are poor at detecting image manipulations (Kasra, Shen, & O’Brien, 2016; Nightingale, Wade, & Watson, 2017). Farid and Bravo (2010), for example, examined whether people can identify discrepancies in image-based cues (e.g., shadows and reflections) that often arise as a result of tampering. Subjects viewed a series of computer-generated scenes consisting of basic geometrical objects. In some scenes, the objects cast accurate (consistent) shadows and reflections, while in other scenes the objects cast impossible (inconsistent) shadows and reflections. When the inconsistencies were blatant—for instance, when shadows ran in opposite directions—subjects identified tampered images with nearly 100% accuracy. Yet when the inconsistencies were subtle—for instance, shadows were a combination of results from two different light positions on the same side of the room—subjects’ performance was close to chance.

More recently, Nightingale et al. (2017) examined people’s ability to detect manipulations of complex, but everyday, real-world scenes. In two online experiments subjects viewed 10 images, half of which were authentic and half of which had been manipulated in one of five ways (e.g., a face was airbrushed or a shadow altered). Although there were differences in subjects’ ability to detect if an image had been manipulated depending on how it had been changed (e.g., image addition and subtractions were better detected than airbrushing, geometrical inconsistences, or shadow inconsistencies), overall performance was close to chance. What’s more, even when people correctly detected a manipulated image, they were often unable to locate where the manipulation was. A related study explored the strategies people use to determine image authenticity (Kasra et al., 2016). Subjects made judgements about the authenticity of images of real-world scenes that were paired with news stories. Consistent with Nightingale et al.’s results, people performed poorly at identifying whether the images had been manipulated. Perhaps more interestingly, subjects reported using nonimage cues, such as the source of the information (e.g., the credibility of the social media platform) or the details in the accompanying story (e.g., the caption provided alongside the image), rather than image-based cues to guide their judgements. In fact, subjects rarely mentioned inconsistencies in lighting and shadows, so it remains unknown if people can make use of such image-based cues even when instructed to do so.

When forgers edit images, they often, inadvertently, create inconsistencies in the physical properties of the scene because 2-D editing of a 3-D scene is difficult. As such, observers might use these inconsistencies to determine whether the image is fake, just as state-of-the-art digital image forensic tools do to determine whether a photo is authentic or not (Farid, 2016; Farid & Bravo, 2010; Kee, O’Brien, & Farid, 2013; O’Brien & Farid, 2012). Although previous research suggests that people might not intuitively use such cues to discriminate between authentic and manipulated images (Kasra et al., 2016; Nightingale et al., 2017), scientists have yet to examine whether image-manipulation detection improves when people are explicitly instructed to do so. We address this question by focusing on shadows (Part 1) and reflections (Part 2), that, in principle, provide observers with a reliable and relatively simple method to verify an image.

## Part 1: Cast shadows

Cast shadows are formed when an opaque object obstructs light and prevents it from illuminating a surface, such as the ground. Because light travels in a straight line, a point in a shadowed region, its corresponding point on the shadow-casting object, and the light source must all lie on a single straight line (Farid, 2016). As such, shadows provide information about the geometry of a 3-D scene and can be used to determine the location of the 3-D light source (Casati, 2004; Farid, 2016; Farid & Bravo, 2010). Assuming linear perspective, then lines in 3-D are imaged as lines in 2-D—that is, the physical laws that constrain the behavior of light in the 3-D world also apply to 2-D images (Farid, 2016; Kajiya, 1986), so when we take photos, or render images from a virtual environment, the interaction of light and the 3-D objects in the scene is captured in the geometry of the 2-D image.^1^

**Fig. 1:**
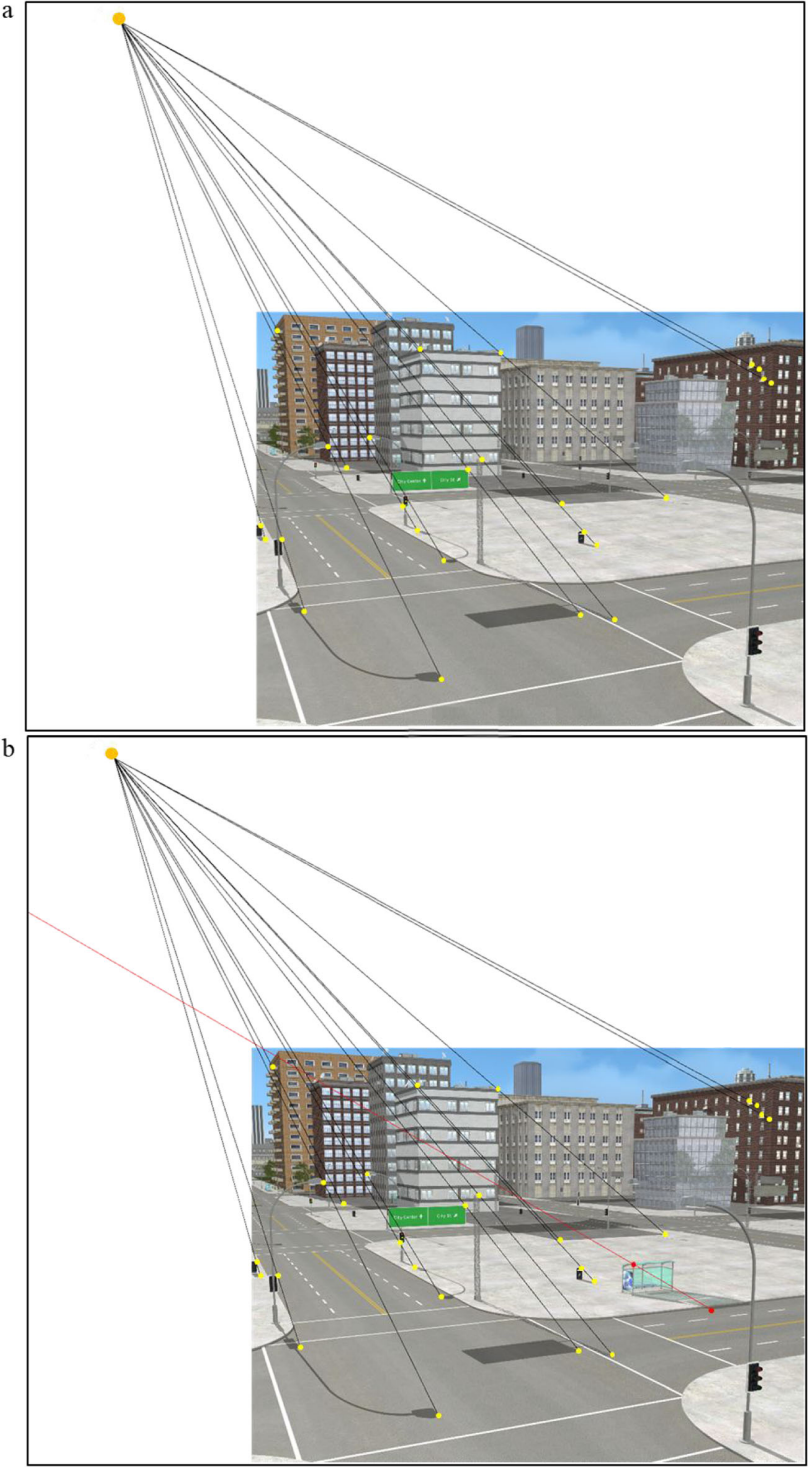
Example of using the shadow-based analysis technique. **a** The lines connecting the corresponding points of the shadows and objects intersect at a single point (yellow dots), indicating that the shadows are consistent with a single light source. **b** The same scene is shown with a bus stop added—the line connecting the bus stop’s shadow and the corresponding point on the object (red dots) does not intersect the scene light source, highlighting an inconsistency. (Color figure online)

The constraint that connects the shadow, the shadow-casting object, and the light source provides an image-based technique for objectively verifying the authenticity of shadows (Farid, 2016; Kee et al., 2013). The scene in Fig. 1a contains shadows that are consistent with a single light source and have not been manipulated. The geometric technique has been applied to objectively demonstrate the authenticity of the shadows in the scene. To use this technique, one can locate any point on a shadow and its corresponding point on the object, then draw a line through them. Repeating this process for as many corresponding shadow and object points as possible reveals the point at which these lines intersect and the exact location of the projection of the light source. In Fig. 1b, a bus stop and its shadow have been taken from another scene where the light source is in a different position and added to the original scene from Fig. 1a. Using the same principle, the line connecting the bus stop’s shadow and the corresponding point on the object does not intersect the scene’s light source. This inconsistency indicates that the image has been manipulated—and demonstrates how shadows can be helpful in detecting forgeries (Farid, 2016; Kee et al., 2013).^2^

We might predict that people can make use of shadow information to help identify image forgeries. Shadows convey important information about the arrangement and spatial position of objects in a scene, and numerous studies show that the human perceptual system makes use of such information to understand the scene (e.g., Allen, 1999; Dee & Santos, 2011; Khang, Koenderink, & Kappers, 2006; Tarr, Kersten, & Bülthoff, 1998). In an early study investigating the perception of inconsistent shadows, people searched for a target cube that was illuminated from a different direction to distractor cubes also present in the display (Enns & Rensink, 1990). Subjects rapidly identified the presence or absence of the target cube, suggesting that the human visual system can process complex visual properties, such as lighting direction, at a preattentive stage of processing. This remarkable ability to perceive shadow information suggests that such information might also help in the detection of image forgeries. Other research, however, suggests that the visual system discounts shadow information in early visual processing (e.g., Ehinger, Allen, & Wolfe, 2016). Essentially, to recognize objects under a wide range of lighting conditions, the visual system prioritizes extraction of the lighting invariant aspects of a scene and filters out shadow information as “noise.” In support of this suggestion, Ehinger et al. found that people were slower to detect changes to shadows than changes to objects even when the shadow changes altered the meaning of the scene. As such, it remains possible that observers will not make use of shadow information to help them to detect image forgeries.

When considering people’s potential ability to make use of shadow information in a given task, it is also important to appreciate that the visual system must determine which shadows are cast by which objects—the shadow correspondence problem (Dee & Santos, 2011; Mamassian, 2004). For stimuli that consist of simple geometric shapes with right-angle features and well-defined shadow regions, matching an object point with its corresponding shadow point can be relatively straightforward—and, accordingly, such stimuli allow for a reasonably accurate estimation of the lighting direction. Yet it is often extremely challenging to match shadow points with corresponding object points in real-world scenes. For example, research suggests that the ability to estimate lighting direction does not generally extend to more complex real-world or computer-generated scenes; although there might be a point at which lighting inconsistencies do become noticeable (Ostrovsky, Cavanagh, & Sinha, 2005; Tan, Lalonde, Sharan, Rushmeier, & O’Sullivan, 2015). Furthermore, it is not known whether the visual system automatically picks up on discrepancies in lighting direction and generates a signal that these should be attended (e.g., the way a single red item among green items might call attention to itself due to a local contrast difference; Lovell, Gilchrist, Tolhurst, & Troscianko, 2009; Rensink & Cavanagh, 2004).

In sum, studies have yet to determine whether people can identify consistent and inconsistent shadows in complex scenes when there are a number of well-defined points between objects and corresponding shadows that, theoretically, make it possible to determine the location of the light source. In the first series of experiments, we aimed to answer this question.

### Experiment 1

#### Method

Subjects and design

A total of 102 subjects (*M* = 25.5 years, *SD* = 9.0, range: 14–57 years; 60 men, 39 women, three chose not to disclose their gender) completed the task online. A further four subjects were excluded from the analyses: three had missing response-time data for at least one response on the task, and one experienced technical difficulties. There were no geographical restrictions, and subjects did not receive payment for taking part, but they did receive feedback on their performance at the end of the task (this was the case for all experiments reported in this paper). The design was within subjects, with each person viewing four computer-generated images, half of which had consistent shadows, and half of which were manipulated to show inconsistent shadows. We measured people’s accuracy in determining whether an image had consistent or inconsistent shadows. A precision-for-planning analysis revealed that 81 subjects would provide a margin of error that is 0.25 of the population standard deviation with 95% assurance^3^ (Cumming, 2012, 2013); this analysis applies to all experiments reported here. All research in this paper was approved by the Psychology Department Research Ethics Committee, working under the auspices of the Humanities and Social Sciences Research Ethics Committee (HSSREC) of the University of Warwick. All participants provided informed consent.

##### Stimuli

To create five different outdoor city scenes, we used a 3-D cityscape model from turbosquid.com and 3-D animation software (Maya^®^, 2016, Autodesk, Inc.).^4^ To represent a real-world outdoor environment lit by the sun, each scene was illuminated by a single distant-point light source.^5^ Each scene included a target object—a lamppost—and its corresponding shadow. To ensure subjects could use the shadow-based analysis technique outlined in the introduction, we included other nontarget objects with corresponding shadows. Recall that when a scene is illuminated by a single source, all of the shadows must be consistent with that light; if any shadow is inconsistent with the light source, then the scene is physically impossible (Farid, 2016; Kee et al., 2013). We rendered each of the five 3-D scenes to generate TIF image files with a resolution of 960 × 720 pixels. For each scene, the light was in front of the camera, but not actually visible within the image. To ensure that the shadows in the 2-D images were physically accurate, and therefore representative of the shadows that people experience in the real world, we rendered the images with raytraced shadows. Raytracing is a type of shadow rendering that calculates the path of individual light rays from the light source to the camera; it produces physically accurate shadows that are like shadows in the real world (Autodesk, 2016). These five scenes comprised our original, consistent image set—each illuminated by a single source and thus containing only consistent shadows.

**Fig. 2:**
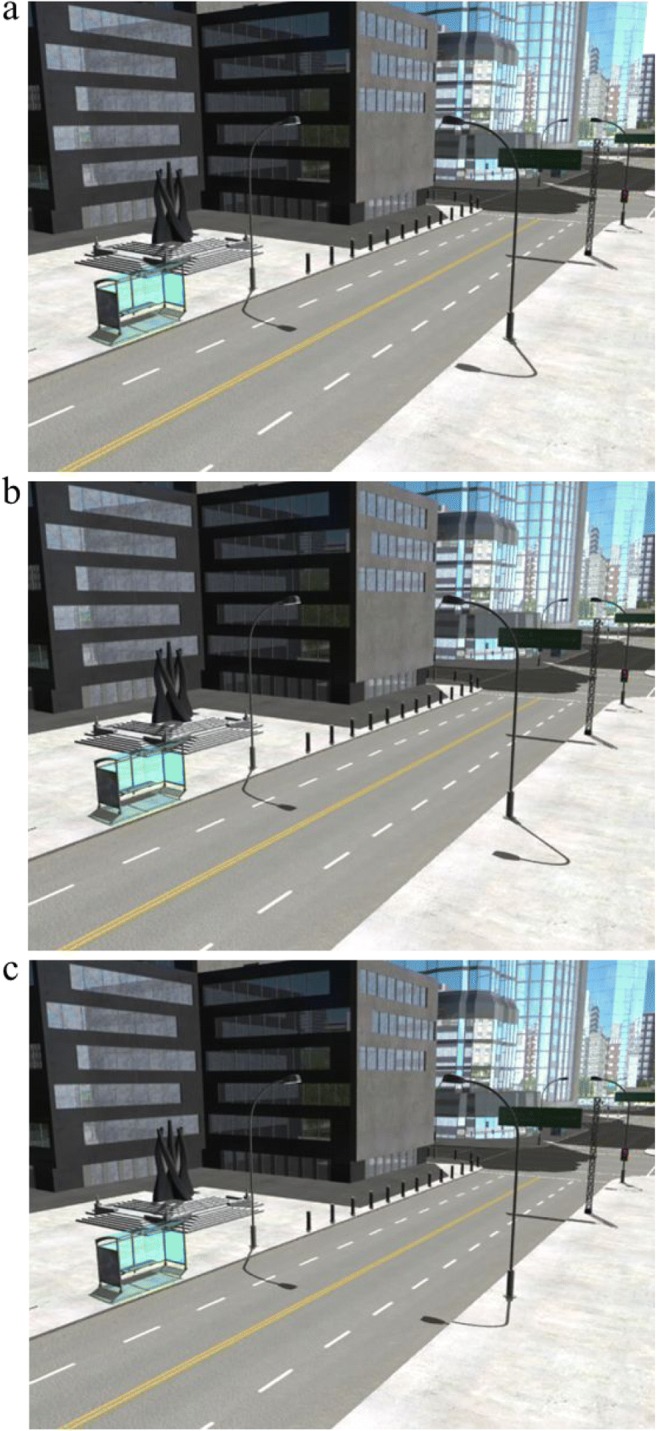
Example of the image-manipulation process. **a** Original scene, with consistent shadows. **b** Left-light position shadow added to the original scene; the shadow of the target lamppost is inconsistent with all of the other shadows in the scene. **c** Right-light position shadow added to the original scene; the shadow of the target lamppost is inconsistent with all of the other shadows in the scene. Each subject saw this city scene just once; they were randomly shown **a**, **b**, or **c**. (Color figure online)

To create the inconsistent-shadow scenes, we rendered each of the five scenes two more times: once with the light moved to the left of its original position (−800 m on the horizontal axis) and once with the light moved to the right of its original position (+800 m on the horizontal axis).^6^ The scene layout remained identical across each version of the scene, yet the three different light positions—original, left, and right—meant that each version had a different shadow configuration. For each of the five scenes, we selected a single lamppost and its corresponding shadow to manipulate. The manipulation process involved three stages completed using GNU Image Manipulation Program^®^ (GIMP, Version 2.8). First, we removed the target lamppost’s shadow in the original version of the scene. Second, we cut the shadow of that same target lamppost from the version of the scene with the light moved left of the original position. Third, we overlaid this shadow onto the original version of the scene. We then repeated stages two and three for the version of the scene with the light moved right of the original position (see Fig. 2). We exported the images as PNGs, which is a lossless format. We repeated this manipulation process for the other four scenes.

Overall, we had three versions of each of the five city scenes to give a total of 15 images. The original version of each scene was used to create our *consistent* shadow image set. The two manipulated versions of each scene were used to create our *inconsistent* shadow image set. The fifth city scene was used as practice (further details on the practice described shortly).

##### Procedure

Subjects were told to assume that “each of the scenes is illuminated by a single light source, such as the sun.” Subjects were given a practice trial before being presented with the four city scenes in a random order. Subjects saw two consistent shadow scenes and two inconsistent shadow scenes; however, they were unaware of this 50:50 ratio. For each scene, to cue subjects’ attention to the target lamppost, they were first shown an almost entirely grayed-out image with only the target lamppost fully visible and highlighted within a red ellipse. After 4 s, the full scene became visible. We also added a small yellow dot on the base of the target lamppost to ensure subjects did not forget which lamppost to consider. Subjects were asked, “Is the lamppost’s shadow consistent or inconsistent with the shadows in the rest of the scene?” They were given unlimited time to select between (a) “Consistent,” (b) “Inconsistent.” They were then asked to rate their confidence in their decision using a 100-point Likert-type scale, from 0 (*not at all confident*) to 100 (*extremely confident*).

After completing the shadow task, subjects were asked a series of questions about their demographics, interest in photography, video gaming experience, and whether they had experienced any technical difficulties while completing the experiment (see Table 6 in Appendix A for exact questions). Finally, subjects received feedback on their performance.

### Results and discussion

For all experiments, we calculated the mean and median response time per image and report these in Table 7 in Appendix A. For all experiments, we followed Cumming’s (2012) recommendations and calculated a precise estimate of the actual size of the effects.

#### Overall accuracy

Can people identify whether scenes have consistent or inconsistent shadows? Overall, a mean 61% of the scenes were correctly classified. Given that there were only two possible response options, chance performance is 50%, thus subjects scored a mean 11 percentage points better than chance. This difference equates to subjects’ performance being a mean 22% better than would be expected by chance alone. Subjects showed a limited ability to discriminate between consistent (75% correct) and inconsistent (46% correct) shadow scenes, discrimination (*d'*) = 0.41, 95% CI [0.22, 0.59].^7^ These findings offer further empirical support for the idea that people have only limited sensitivity to lighting inconsistencies (e.g., Farid & Bravo, 2010; Ostrovsky et al., 2005). Thus it appears that subjects did not use the information available within the scene to work out the answer objectively. Furthermore, they showed a bias towards accepting the shadow scenes as consistent response bias (*c*) = 0.29, 95% CI [0.20, 0.38]. Presumably, our subjects had a relatively conservative criterion for judging that shadows were inconsistent with the scene light source and typically accepted them as consistent.

#### Image metrics and individual factors

Next, we tested whether people’s accuracy on the shadow task was related to the difference between the position of the projected light source for the scene and the projected light source for the inconsistent shadow. To achieve this, we calculated the shortest distance between the projected light position for the scene and a line connecting the target lamppost with its inconsistent shadow. In addition, we checked whether two properties of the image itself affected people’s accuracy on the task: (1) whether the light position had moved left or right of the original light position, and (2) the location of the scene light source. Furthermore, to determine whether individual factors played a role in identifying consistent and inconsistent shadows, we gathered subjects’ demographic data and details about their interest in photography and video gaming. On the shadow task, we also asked subjects to rate their confidence for each of their decisions and recorded their response time.

We conducted exploratory analyses to determine how each factor influenced subjects’ performance by running two generalized estimating equation (GEE) analyses—one for the inconsistent shadow scenes and one for the consistent shadow scenes. Specifically, we conducted a repeated-measures logistic regression with GEE because our dependent variables were binary with both random and fixed effects (Liang & Zeger, 1986). The results are shown in Table 1.

**Table 1:** Results of the GEE binary logistic-regression models to determine variables that predict accuracy in the shadow task

Predictor	Inconsistent			Consistent		
	*B*	*OR* [95% CI]	*p*	*B*	*OR* [95% CI]	*p*
Confidence	0.00	1.00 [0.98, 1.01]	.54	0.01	1.01 [0.99, 1.03]	.16
Video gaming = Frequent (at least once or twice a week)	0.68	1.97 [1.04, 3.72]	.04	0.02	1.02 [0.47, 2.20]	.96
Response time	0.01	1.01 [0.98, 1.03]	.59	0.00	1.00 [0.98, 1.03]	.84
Gender = Female	−0.16	0.86 [0.44, 1.66]	.64	−0.03	0.97 [0.42, 2.24]	.94
Interest in photography = Interested	0.21	1.23 [0.60, 2.52]	.57	−0.12	0.89 [0.38, 2.07]	.78
Distance to light source	0.03	1.03 [0.99, 1.09]	.16	−0.02	0.98 [0.97, 0.99]	<.001
Light position = Left	0.20	1.23 [0.73, 2.07]	.45	–	–	–
Distance from scene light source to inconsistent constraint	−0.01	0.99 [0.98, 1.00]	.15	–	–	–

*Note.* CI = confidence interval. *B* and odds ratios (*OR*) estimate the degree of change in accuracy associated with one unit change in the independent variable. An odds ratio of 1 indicates no effect of the independent variable on accuracy; values of 1.5, 2.5, and 4.0 are generally considered to reflect small, medium, and large effect sizes, respectively (Rosenthal, 1996). The category order for factors was set to descending to make the reference level zero. The reference groups are video-game playing = infrequent (never/less than once a month/about once a month/a couple of times a month); gender = male; interest in photography = not interested; light position = right. Response time, confidence, distance of light source from the scene, and angle difference were added as continuous variables. The three subjects who chose not to disclose their gender were excluded from these analyses, leaving a total sample of *n* = 99. The light position and distance from scene light source to inconsistent constraint predictor variables were not applicable in the consistent shadow scenes.

The distance between the scene light source and inconsistent shadow constraint did not predict accuracy on the task. This result suggests that people either are not aware that they can use this geometrical image-based technique for objectively verifying the authenticity of shadows or that they make errors when trying to apply this technique. For example, the shadow correspondence problem (Dee & Santos, 2011; Mamassian, 2004) might limit the extent to which subjects were able to accurately estimate the position of the scene light source. Video-game playing was the only variable in the model that had an effect on the likelihood of responding correctly. Those who play video games frequently (at least once or twice a week) were more likely to correctly identify inconsistent shadow scenes than those who do not. At first glance, this finding seems consistent with previous research showing that video gamers outperform non-video-gamers across a range of perceptual measures (for a review, see Green & Bavelier, 2012). Yet a more recent review of these studies highlights a number of methodological flaws in the research (Simons et al., 2016). These flaws, along with the exploratory nature of the analysis in the current study, limit the extent to which we can draw any firm conclusions about the effect of video gaming on visual tasks.

For the consistent shadow scenes, the distance of the projected light source from the scene had a small effect on the likelihood of responding correctly. Scenes in which the projection of the light onto the image plane was closer to the center of the image were more likely to be identified as consistent than scenes in which the light was further from the center. Subjects might have been better able to determine the accuracy of shadows in a scene when the light source was more readily available to use as a guide. Perhaps, then, our subjects were able to make use of the shadow-based analysis technique, but only when it was relatively easy to calculate the location of the projected light source.

For each of the four scenes in our experiment, the projection of the light source was beyond the image plane. Therefore, applying the geometric shadow-based analysis technique with our stimuli required people to use information outside of the image plane. It is possible that this is a difficult task to perform perceptually and that, instead, people tended to more frequently rely on in-plane image cues. We tested this suggestion by running a second GEE analysis for the inconsistent shadow scenes. In this second analysis, we examined whether a new variable measuring the rotation, in degrees, between the consistent shadow position and the inconsistent shadow position (computed on the image plane) was related to accuracy on the task. We included this angle difference measure in the second GEE analysis in place of the variable that measured the distance between the scene light source and inconsistent shadow constraint. All other variables in the model remained the same.

As shown in Table 2, this time two variables had an effect on the likelihood of responding correctly. First, replicating the result of the first model, those who play video games frequently were more likely to correctly identify inconsistent shadow scenes than those who do not. Second, inconsistent shadows positioned further from the correct position were more likely to be associated with accurate responses than inconsistent shadows positioned closer to the correct position were. This finding suggests that there might be a discernible point at which the inconsistent shadow becomes different enough from its correct position to make the inconsistency noticeable—lending support to the notion of a perceptual threshold for detecting lighting inconsistencies (Lopez-Moreno, Sundstedt, Sangorrin, & Gutierrez, 2010; Tan et al., 2015). In other words, our subjects appeared to hold a basic understanding of where an object’s shadow must cast to be consistent with the light source, but their understanding was imprecise.

**Table 2:** Results of the follow-up GEE binary logistic regression model to determine variables that predict accuracy in the shadow task

Predictor	Inconsistent		
	*B*	*OR* [95% CI]	*p*
Confidence	0.00	1.00 [0.98, 1.01]	.50
Video gaming = Frequent (at least once or twice a week)	0.74	2.09 [1.08, 4.01]	.03
Response time	0.00	1.00 [0.98, 1.03]	.71
Gender = Female	−0.19	0.82 [0.42, 1.61]	.57
Interest in photography = Interested	0.18	1.19 [0.57, 2.48]	.64
Distance to light source	0.00	1.00 [0.99, 1.01]	.68
Light position = Left	0.17	1.18 [0.70, 2.00]	.54
Angle difference	0.03	1.03 [1.00, 1.05]	.04

*Note.* CI = confidence interval. *B* and odds ratios (*OR*) estimate the degree of change in accuracy associated with one unit change in the independent variable. An odds ratio of 1 indicates no effect of the independent variable on accuracy; values of 1.5, 2.5, and 4.0 are generally considered to reflect small, medium, and large effect sizes, respectively (Rosenthal, 1996). The category order for factors was set to descending to make the reference level zero. The reference groups are video-game playing = infrequent (never/less than once a month/about once a month/a couple of times a month); gender = male; interest in photography = not interested; light position = right. Response time, confidence, distance of light source from the scene, and angle difference were added as continuous variables. The three subjects who chose not to disclose their gender were excluded from the analysis, leaving a total sample of *n* = 99.

Overall, subjects were slightly more likely to identify the inconsistent shadows when the angle difference from the correct shadow location was larger compared with when it was smaller. Yet the experimental design meant that there were only eight inconsistent shadow scenes and thus only eight angle differences to examine. In Experiments 2a and 2b, to more precisely estimate the perceptual threshold for identifying lighting inconsistencies, we asked subjects to rotate a target shadow to the position that they thought was consistent with the lighting of the scene.

### Experiments 2a and 2b

The results of Experiments 2a and 2b largely replicate those of Experiment 1, except using a different experimental paradigm. Thus, for brevity, we present full details of Experiments 2a and 2b in Appendix B and summarize the findings here.

In Experiment 2a, subjects were able to change the shadow rotation 360° about the base of the target lamppost; their task was to place the shadow in the position that they believed to be consistent with the other shadows in the scene. Even with this high level of control over the shadow position, subjects were willing to rotate the shadow to a relatively wide range of positions that were inconsistent with the scene lighting—51% of the shadows were positioned between −10° and +10° of the consistent position, 95% CI [46%, 56%]. Although there were differences by scene, overall a mean 20% more shadows were positioned to the left than to the right of the correct location, *M*_diff_ 95% CI [12%, 28%].

In Experiment 2b, subjects could both rotate the shadow and change the size of the shadow. The results were similar to those in Experiment 2a, with 46% of shadows positioned between ±10° of the consistent position, 95% CI [41%, 51%]. Replicating Experiment 2a, collapsed across the four scenes, subjects positioned 16% more of the shadows to the left of the correct position than to the right, *M*_diff_ 95% CI [7%, 25%]. In sum, allowing subjects to adjust the size of the target shadow in Experiment 2b made virtually no difference to the pattern of results.

Overall, the results from Experiments 2a and 2b indicate that subjects frequently make imprecise judgements about where shadows must be positioned to be consistent with a single light source. It is important to note, however, that each target shadow in Experiments 2a and 2b was simply the correct one for the given scene rotated around the base of the object. That is, the manipulations were made on the image plane rather than in the 3-D environment. As such, incorrect shadows were also inconsistent with the casting object in terms of sizes and angles between the lamp and the pole parts of the object/shadow. Therefore it is possible that being able to change the scale of the target shadow did not prevent subjects using the shape of the shadow as a cue. If so, our results might still overestimate people’s ability on the task. To examine this possibility, in Experiment 3, using the 3-D environment, we generated different versions of the target shadow that were inconsistent with the scene light source in terms of both orientation and shape. Importantly though, in Experiment 3, each inconsistent shadow option was physically plausible with respect to a single light source (albeit not the scene light source) in terms of its size and angle.

### Experiment 3

#### Method

##### Subjects and design

A total of 114 subjects (*M* = 25.2 years, *SD* = 8.4, range: 14–52 years; 48 women, 62 men, four chose not to disclose their gender) completed the task online. Five additional subjects were removed because they experienced technical difficulties. We used a within-subjects design.

##### Stimuli

**Fig. 3:**
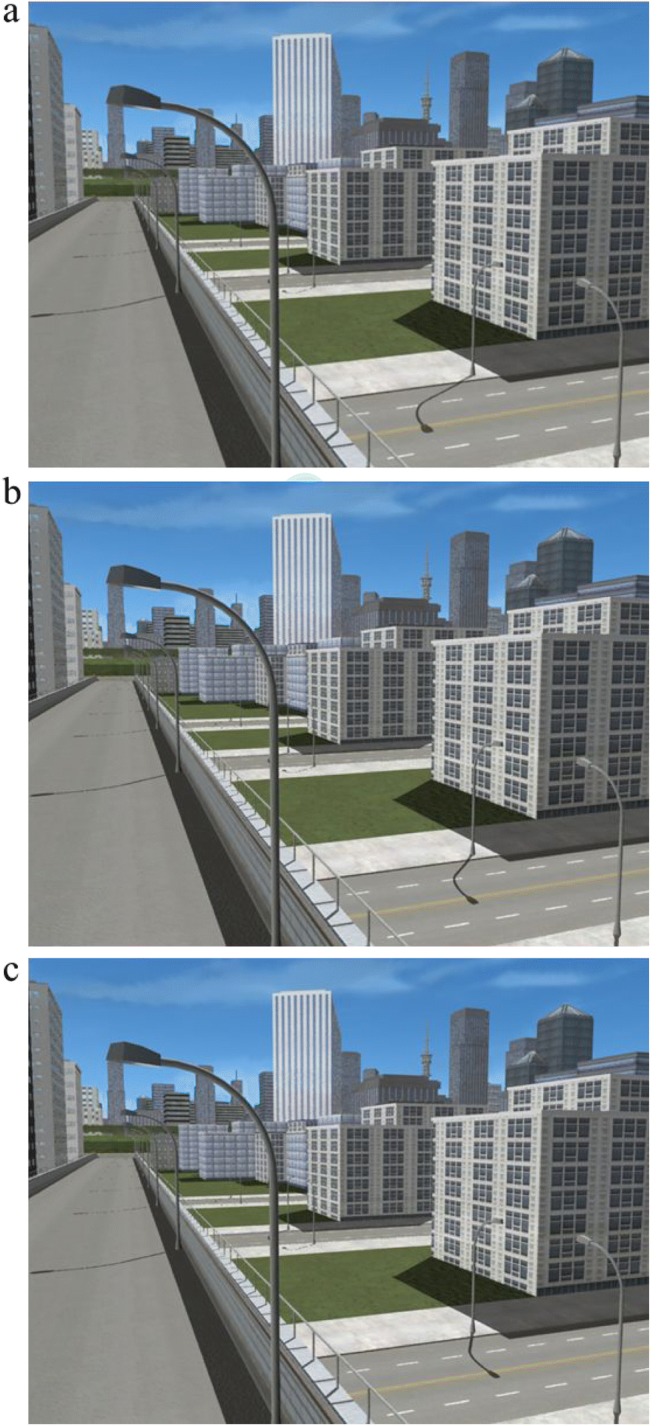
Example versions of Scene 3. **a** Version 4, inconsistent. **b** Version 11, consistent. **c** Version 14, inconsistent

We used the same city scenes as in the previous experiments. This time, however, we created 21 versions of each scene, each version with the objects in an identical position, but with 21 different light positions. In the consistent version, the target lamppost’s shadow was created by the same light source as the rest of the scene. In the other 20 inconsistent versions, we created a second light source that only cast a shadow for the target lamppost. By changing the position of the second light source only, we created 20 versions of the scene in which the shadow for the target lamppost was inconsistent with the shadow configuration for the rest of that scene—but physically consistent with being cast by the target lamppost. For 10 of the inconsistent versions of the scene, we moved the second light source in 10 equal increments of 200 m to the left of the original light position. For the other 10, we moved the second light source in 10 equal increments of 200 m to the right of the original light position. As a result, we created 21 versions of each of the five scenes: one with consistent lighting for all objects in the scene—including the target lamppost—and 20 with consistent lighting for all objects except the target lamppost. The versions of the scene were numbered from 1 to 21, with the consistent version of the scene always number 11. Versions 10 to 1 were inconsistent, with the target shadow moving incrementally further to the left of the consistent version, while versions 12 to 21 were inconsistent, with the target shadow moving incrementally further to the right of the consistent version (see Fig. 3 for examples).

We developed a program in HTML to randomly select one of the 21 versions of the scene to display. As well as this randomly selected version, subjects were able to scroll through a sequence of another 10 consecutive versions of that same scene—crucially, the sequence always included the consistent version. To illustrate, consider, for example, that the program randomly selects Version 1, the subject would be able to scroll through Versions 1 to 11 of the scene. Or, to consider another example, if the program randomly selects Version 15, then the subject will be able to scroll through Versions 5 to 15 of the scene. Having generated the sequence, the program randomized which of the 11 versions to display first, thus ensuring that subjects did not always start at the extreme end of a sequence. Subjects used the left and right arrow keys on the keyboard to scroll through the 11 versions.

##### Procedure

The procedure was the same as in Experiment 1, with one exception: Subjects scrolled through the 11 versions of each scene rather than deciding whether the shadows in each scene were (a) “Consistent” or (b) “Inconsistent.” We asked subjects to select the version of the scene in which the shadow of the target lamppost was consistent with the other shadows in the scene.

### Results and discussion

Overall accuracy

**Fig. 4:**
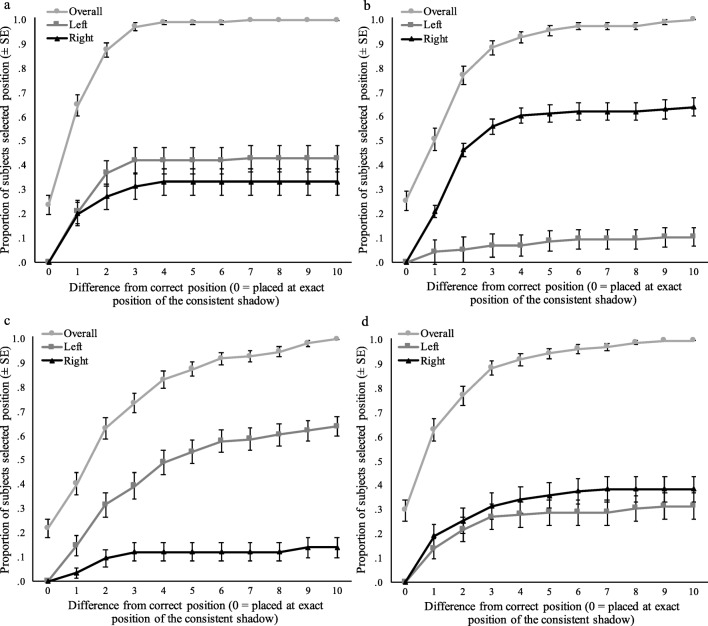
Cumulative proportion of responses made by each version of the scene for (**a**) Scene 1, (**b**) Scene 2, (**c**) Scene 3, and (**d**) Scene 4. The light-gray line with circle markers shows the overall proportion of responses made by each inconsistent version of the scene—these are cumulative, and therefore a difference of 1 includes subjects selecting Versions 10, 11, or 12 of the scene. The black line with triangle markers shows the cumulative proportion of subjects selecting each version of the scene to the right of the consistent shadow position responses. The dark-gray line with square markers shows the cumulative proportion of subjects selecting each version of the scene to the left of the consistent shadow position responses. Error bars represent standard error of the mean.

Subjects’ performance on the shadow task can be classified in different ways. Taking a conservative approach, we defined an accurate response to be only when subjects selected the (single) consistent version of the scene. Collapsed across the four scenes, the consistent version was selected a mean 25% of the time, 95% CI [21%, 29%]. Given that there were 11 possible response options in the task chance performance is 9%, thus subjects scored a mean 16 percentage points better than chance, 95% CI [12%, 20%]. This difference equates to subjects’ performance being a mean 178% better than would be expected by chance alone. Taking a more lenient approach and defining an accurate response by including one version either side of the consistent shadow position—that is, when versions 10, 11, or 12 were selected—a mean 55% of shadows were positioned correctly, 95% CI [50%, 59%]. Replicating the findings from Experiments 2a and 2b (see Appendix B), Fig. 4 shows that subjects were least accurate for Scene 3, with a mean 40% selecting Versions 10, 11, or 12, 95% CI [31%, 49%]. In contrast to the previous experiments, however, subjects were most accurate for Scene 1, with a mean 65% selecting Versions 10, 11, or 12, 95% CI [56%, 74%]. There is no immediately obvious reason as to why subjects did relatively well on Scene 1 in Experiment 3. Speculatively, it is possible that the shape of the target shadow in Experiments 2a and 2b actually made the inconsistent positions seem more plausible rather than less plausible in Scene 1.

#### Preference for shadows to the left or right

In contrast to the results of Experiments 2a and 2b (see Appendix B), collapsing across all four scenes, the shadows were equally likely to be positioned to the left or to the right of the correct location, M_diff_ = 0%, 95% CI [−8%, 7%]. Yet as Fig. 4 shows, there was still variation by scene. In line with our previous experiments, subjects were more likely to position the target shadow left of the correct position in Scene 3. And again, in Scene 2, subjects were more likely to position the target shadow right of the correct position. This time, in both Scenes 1 and 4, a similar proportion of subjects selected a target shadow to the left of its correct location as to the right.

Overall, the pattern of results across our four shadow experiments was largely consistent. Most importantly, the experiments suggest that people have a limited ability to identify consistent and inconsistent shadows. This finding is somewhat surprising considering that subjects viewed scenes in which there was sufficient information to determine the answer objectively. Next, we consider the extent to which people make use of reflections to identify authentic and manipulated scenes.

## Part 2: Reflections

Shortly after the November 2015 Paris terrorist attacks, a doctored photo depicting an innocent man—Veerender Jubbal—as one of the attackers circulated online and in newspapers (Butterly, 2015; Rawlinson, 2015). Although many major news outlets were fooled by the image, the manipulation left several prominent clues that the image was a fake. Crucially, when Jubbal photographed his reflection, he was standing straight-on to the mirror, which means that the camera used to capture the photo must also be visible in the reflection. In the authentic version of the photo, the iPad^®^ used to capture the photo can be seen clearly in the reflection (Jubbal, 2016). Yet in the manipulated version of the image, the forgers replaced the iPad^®^ with a Qur’an, thus making it a geometrical impossibility for the image to have been captured. Detecting this basic inconsistency in the geometry of the reflection could have prevented an innocent man from becoming a suspect. To what extent, then, can people use reflections to help to identify whether photos are authentic or manipulated? We explore this question next.

A small number of studies have explored what people understand about mirror reflections^8^ (Bertamini, Spooner, & Hecht, 2003; Bianchi & Savardi, 2012; Hecht, Bertamini, & Gamer, 2005; Lawson, 2010; Lawson & Bertamini, 2006; Muelenz, Hecht, & Gamer, 2010). In one study using a bird’s-eye-view diagram of a room, subjects predicted when a target would first become visible in a mirror (Croucher, Bertamini, & Hecht, 2002). In one scenario, for example, subjects indicated the point at which a character who walks across the room on a path parallel to the surface of a mirror on the opposite wall would first see their reflection in that mirror. Across a range of these scenarios, subjects made consistent *early errors*, predicting that the character would be able to see their reflection before reaching the edge of the mirror (a physically impossible occurrence). These findings suggest that people’s understanding of reflection is limited and biased.

Yet, if people have such a limited understanding of reflections, why can they make effective use of mirrors in everyday life—for instance, when driving or checking their appearance? One possibility is that when using real mirrors the availability of perceptual information allows people to use them effectively. Typically, such information is not available in experimental reflection tasks (Bertamini et al., 2003; Croucher et al., 2002; but see Lawson & Bertamini, 2006), so people might rely on perceptual biases instead. For example, Muelenz et al.’s (2010) findings suggest that people hold a perceptual outward bias that causes them to mentally rotate the mirror reflection of the world to make it more orthogonal (at a right angle) with respect to their line of sight. As a result, the mirror reflection appears further away from the observer than it would in reality.

**Fig. 5:**
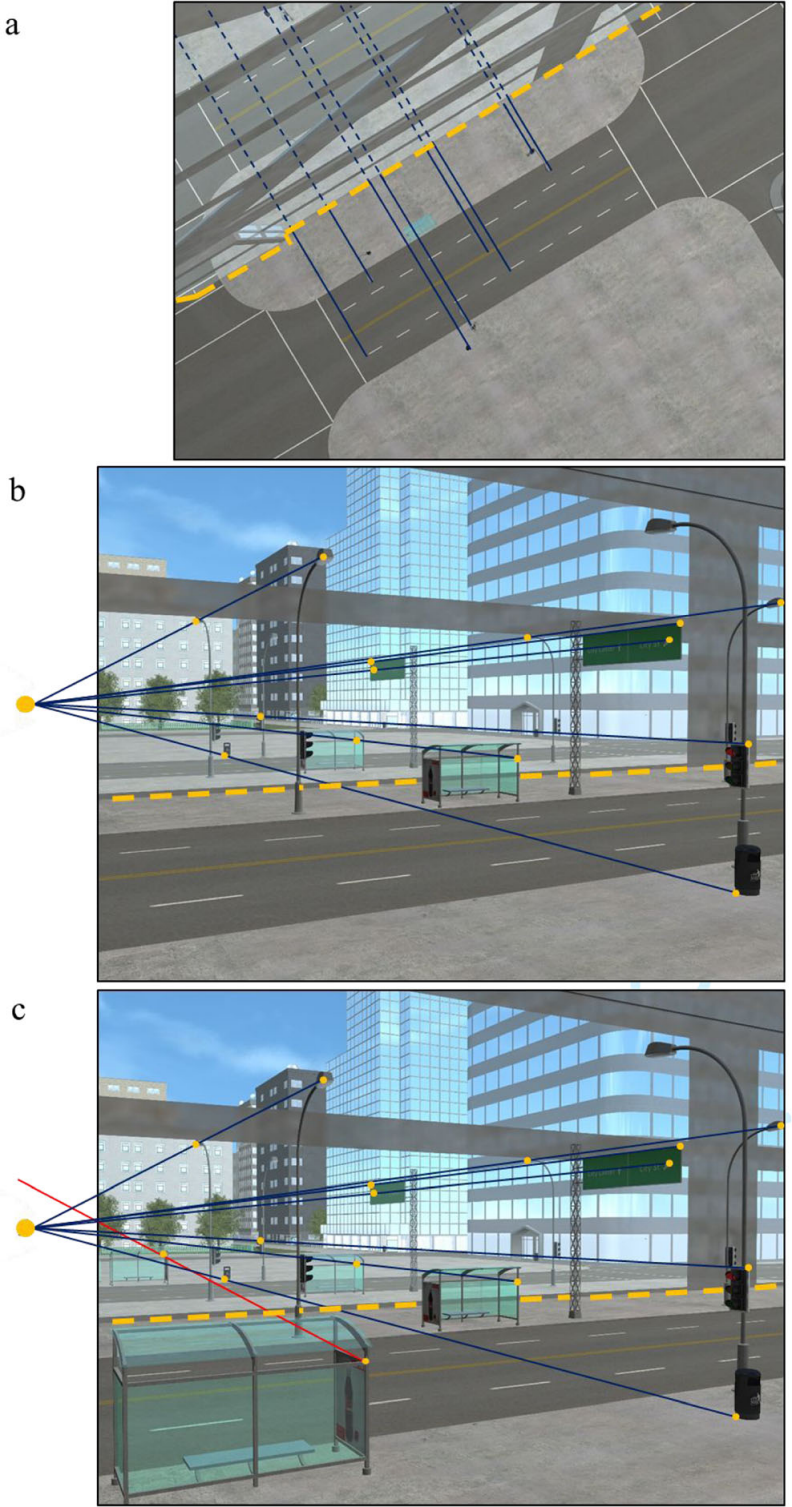
Example of the geometrical information available in images. **a** The scene is shown from a bird’s-eye view. The dashed-yellow line corresponds to where the mirrored plane intersects the sidewalk. The geometric relationship between objects and reflections of those objects is constrained by the optical law of reflection. As a result, the blue lines drawn connecting the objects with their reflections are parallel to one other and perpendicular to the mirror surface. For clarity, the lines appear solid in the world and become dashed as they cross into the reflection of the world. **b** The same scene is shown from a different perspective; here, owing to perspective projection, the blue lines are no longer parallel, but instead converge to a single vanishing point. **c** Again, the same scene is shown, but the bus stop on the left, and its reflection, has been added. The red line connecting the bus stop with its reflection does not intersect the scene’s reflection vanishing point and highlights an inconsistency. (Color figure online)

In 2-D scenes, one piece of information that might prove useful and help to override reliance on the outward bias is the reflection vanishing point (Montague, 2013). Reflections adhere to a basic law of optical physics: A smooth surface will reflect the light at the same angle that it hits the surface (Hecht & Zajac, 1974; Ronchi, 1970). As shown in Fig. 5a, a result of this optical constraint means that, from a bird’s-eye view, imaginary parallel lines connect points on the real object in front of the mirror with the same points on the object’s reflection behind the mirror (Farid, 2016; O’Brien & Farid, 2012). Owing to linear perspective projection, when viewing that same scene but from the viewpoint shown in Fig. 5b, the lines that connect object points and their corresponding points in the reflection will converge to a single point—the reflection vanishing point. Consequently, a geometric-based analysis can be used to objectively verify where the reflection of an object in the world should appear in a mirror. Furthermore, adding fake reflections into a photo, or manipulating a photo that contains reflections, can create inconsistencies that can be identified using geometric-based analysis. If a line connecting a point on an object and its corresponding point in the reflection does not intersect the reflection vanishing point, it highlights an inconsistency (O’Brien & Farid, 2012). As shown in Fig. 5c, the bus stop’s reflection is inconsistent with the reflection vanishing point that is consistent with the rest of the scene, indicating that some manipulation has occurred. This reasonably simple analysis based on the geometric relationship between objects and their reflections is used in digital image forensics to help identify fakes (O’Brien & Farid, 2012). Although this analysis uses reflections and the reflection vanishing point, it works using the exact same geometric constraint as with shadow analysis.

What is still unknown, however, is whether people can identify the presence of consistent and inconsistent reflections in complex scenes when there is sufficient information available that, theoretically, makes it possible to determine the location of the reflection vanishing point.

### Experiment 4

#### Method

##### Subjects and design

A total of 79^9^ subjects (*M* = 26.6 years, *SD* = 10.8, range: 13–68 years, 39 men, 38 women, two chose not to disclose their gender) completed the study online. Five additional subjects were removed: four experienced technical difficulties, and one had missing response time data for at least one response on the task. We used a within-subjects design.

##### Stimuli

We created five different outdoor city scenes from the same 3-D cityscape model that was used to create the shadow scenes in Experiments 1–3. All scenes were rendered as TIF files at a resolution of 960 × 720 pixels. Each of the five scenes included a flat, smooth reflective surface (part of a building frontage). In each scene, the target object was a street sign placed adjacent to the reflective surface so that the sign and its reflection were both visible. To ensure that subjects could use geometric analysis to locate the reflection vanishing point, we made a number of other nontarget objects and their corresponding reflections visible in the scene. The scenes were rendered without shadows (by using ambient lighting, but no directional light) to ensure that we did not provide subjects with any additional cues that would influence their ability on the reflection task. These five scenes were the originals with consistent reflections.

**Fig. 6:**
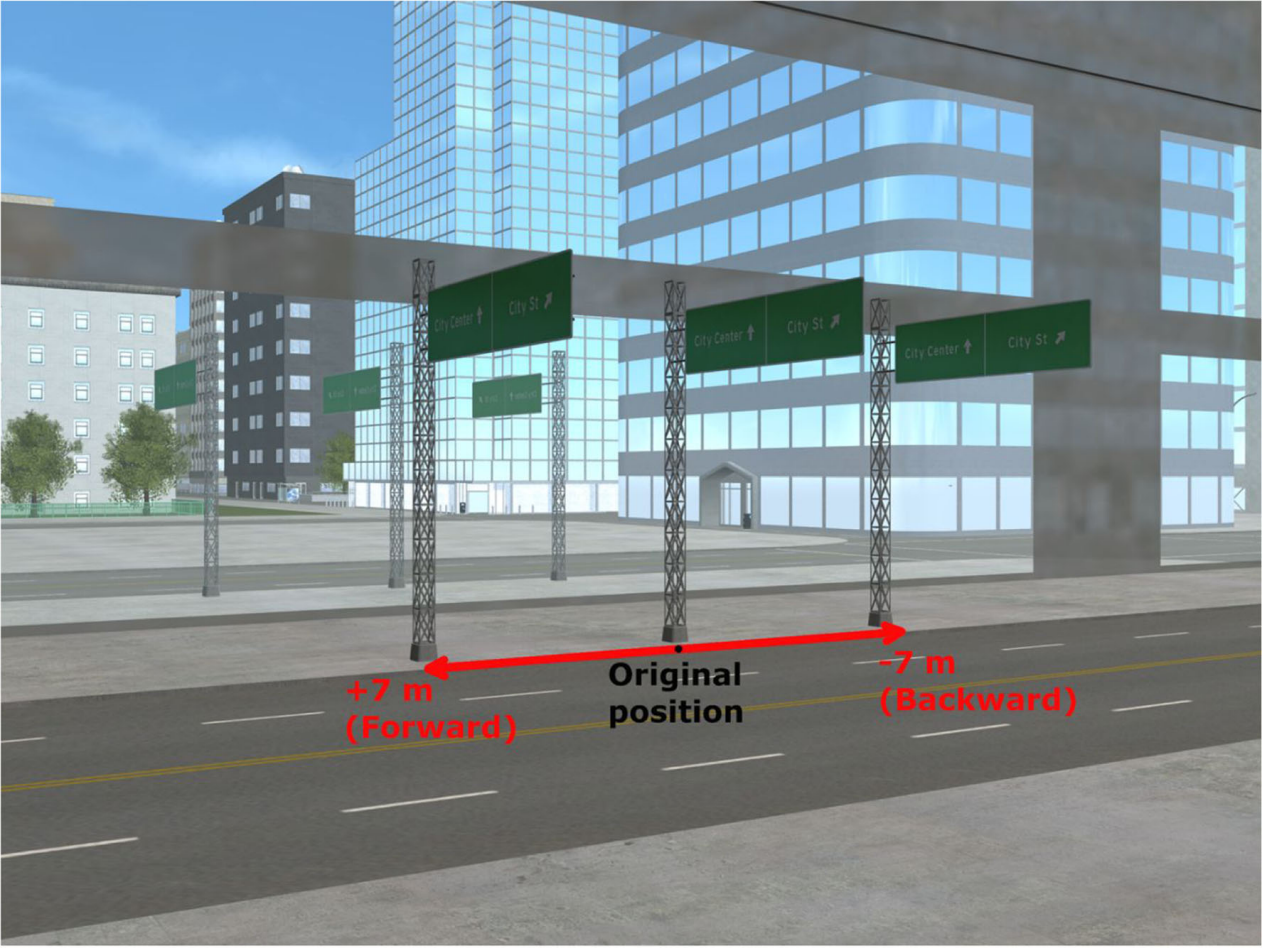
Example showing the movement of the original street sign and its reflection forward and backward parallel to the reflection plane.

**Fig. 7:**
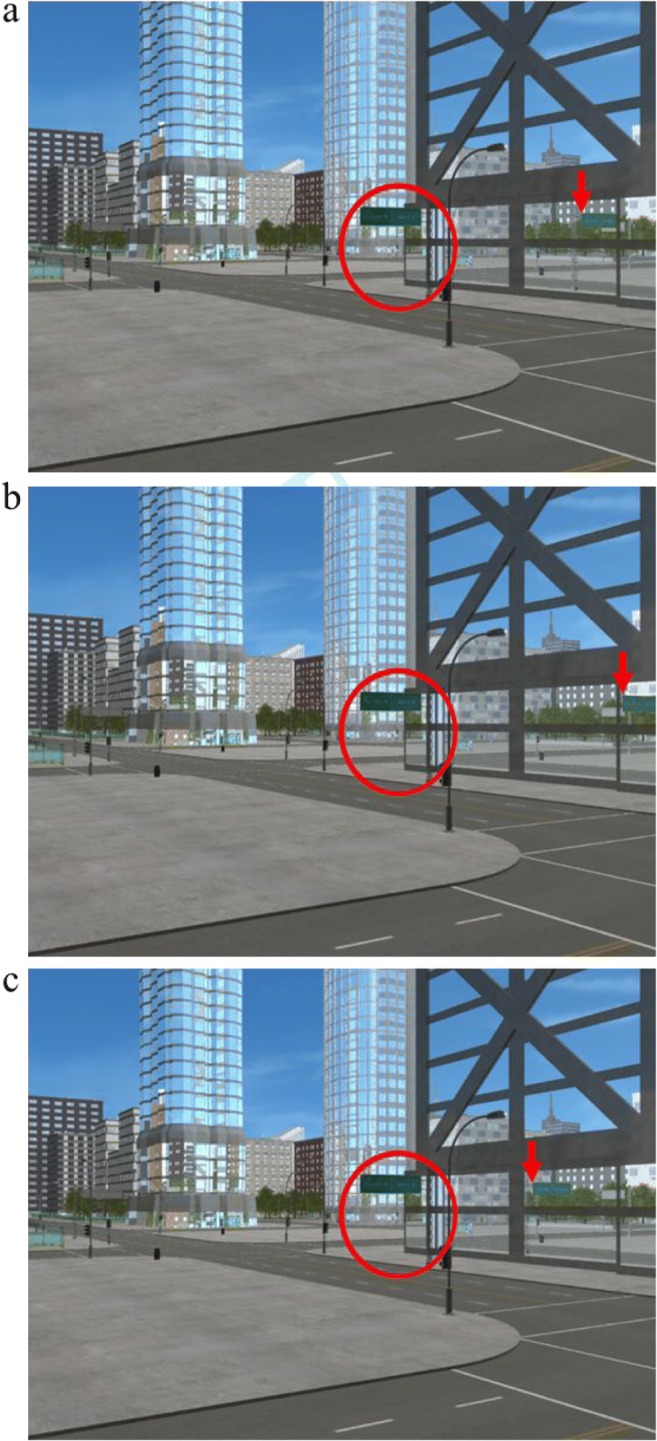
Example of the consistent and two inconsistent versions of a scene. **a** Original image with consistent reflections. **b** Forward inconsistent reflection image with the target street sign reflection moved forward of the consistent position. **c** Backward inconsistent reflection image with the target street sign reflection moved backward of the consistent position. Each subject saw this city scene just once, and they were randomly shown **a**, **b**, or **c**. In the example images, the target street sign is shown in a red circle, and a red arrow indicates the location of the street sign’s reflection. (Color figure online)

To create our inconsistent reflection scenes, we began by rendering each scene two more times; once with the street sign moved forward relative to the original street sign position (+7 m on the axis that is parallel to the reflection plane) and once with the street sign moved backward relative to the original street sign position (−7 m on the axis that is parallel to the reflection plane; see Fig. 6). We then manipulated the images using GNU Image Manipulation Program^®^ (GIMP, Version 2.8). First, we removed the street sign’s reflection in the original scene, importantly, the original street sign itself remained in the scene. Second, we cut the reflection of the street sign from one of the other scenes with the street sign moved forward or backward. We then overlaid this reflection onto the original scene (see Fig. 7 for an example of the consistent and inconsistent versions of a scene).

Overall, we produced three versions of each of the five city scenes (15 images in total). The original, nonmanipulated version of each of these scenes was used to create our consistent reflection image set. The two manipulated versions of each scene were used to create our inconsistent reflection image set. Subjects saw two consistent-reflection and two inconsistent-reflection images presented at random, but always in a different city scene. The fifth city scene was used as a practice.

##### Procedure

We used the same procedure as Experiment 1, with the following changes: (1) We cued subjects’ attention to the target street sign on which they needed to base their response, and (2) we asked, “Is the street sign’s reflection consistent or inconsistent with the other reflections in the scene?”

#### Results and discussion

##### Overall accuracy

Overall, a mean 50% of the scenes were classified correctly, with chance performance being 50%. Subjects’ ability to distinguish between consistent (42% correct) and inconsistent (58% correct) reflections was not reliably greater than zero, *d'* = −0.01, 95% CI [−0.23, 0.21]. Furthermore, subjects showed a bias toward saying that reflections were inconsistent, *c* = −0.15, 95% CI [−0.25, -0.06]. These results indicate that subjects found it extremely difficult to determine whether the reflection of the target object was consistent or inconsistent with the other reflections in the scene. Furthermore, the results suggest that subjects did not make use of the geometrical information in the scene to compute the reflection vanishing point and objectively determine the answer. Instead, it is possible that subjects had incorrect beliefs about reflections and perhaps relied on these to make a subjective judgement about the consistency or inconsistency of the reflections in the scene (see, e.g., Bertamini et al., 2003; Croucher et al., 2002).

##### Image metrics and individual factors

We checked whether three properties of the image itself affected people’s accuracy on the task. One image property was simply whether the reflection had moved forward or backward relative to the consistent reflection position (see Fig. 7). The second image property was the distance from the center of the image to the reflection vanishing point. The third image property was an angle measurement—the rotation, in degrees, from the scene’s reflection vanishing point to the reflection vanishing point for the target object and its inconsistent reflection. In addition, to determine whether individual factors played a role in identifying consistent and inconsistent reflections, we included the same individual factors as used in the GEE models in Experiment 1.

We conducted exploratory analyses to check how each factor influenced subjects’ performance by running two GEE analyses—one for the inconsistent reflection scenes and one for the consistent reflection scenes. The results are shown in Table 3.

**Table 3:** Experiment 4. Results of the GEE binary logistic models to determine variables that predict accuracy in the reflection task

Predictor	Inconsistent			Consistent		
	*B*	*OR* [95% CI]	*p*	*B*	*OR* [95% CI]	*p*
Reflection position = forward	0.82	2.26 [1.08, 4.74]	.03	–	–	–
Confidence	0.01	1.02 [1.00, 1.03]	.04	−0.01	0.99 [0.98, 1.01]	.24
Gender = female	0.71	2.04 [1.01, 4.10]	.05	−0.10	0.91 [0.46, 1.78]	.78
Vanishing point distance	−0.05	0.95 [0.89, 1.01]	.12	0.03	1.03 [0.97, 1.11]	.34
Interest in photography = interested	−0.61	0.54 [0.25, 1.16]	.12	−0.21	0.81 [0.41, 1.59]	.54
Video gaming = frequent (at least twice a month)	0.47	1.59 [0.80, 3.18]	.19	0.40	1.49 [0.79, 2.83]	.22
Angle difference	−2.22	0.11 [0.00, 3.96]	.23	–	–	–
Response time	0.00	1.00 [1.00, 1.01]	.41	0.00	1.00 [0.99, 1.01]	.79

*Note.* CI = confidence interval. *B* and odds ratios (*OR*) estimate the degree of change in accuracy associated with one unit change in the independent variable. An odds ratio of 1 indicates no effect of the independent variable on accuracy; values of 1.5, 2.5, and 4.0 are generally considered to reflect small, medium, and large effect sizes, respectively (Rosenthal, 1996). The category order for factors was set to descending to make the reference level zero. The reference groups are reflection position = back; video-game playing = infrequent (never/less than once a month/about once a month); gender = male; interest in photography = not interested. Response time, confidence, reflection vanishing point distance, and angle difference were added as continuous variables. The two subjects who chose not to disclose their gender were excluded from these analyses, leaving a total sample of *n* = 77. The reflection position and angle difference predictor variables were not applicable in the consistent reflection scenes.

Three variables had an effect on subjects’ ability to accurately identify inconsistent reflection scenes: reflection position, confidence, and gender. Scenes in which the reflection was moved forward from its consistent position were more likely to be identified as inconsistent compared with scenes in which the reflection was moved backward from its consistent position. Indeed, the inconsistent reflections that appeared further away rather than closer to the observers’ viewpoint were more likely to be incorrectly accepted as consistent. One possibility is that this result is simply an effect of perspective projection from the 3-D world to the 2-D image. Although the street sign was moved equally in the forward and backward conditions in the 3-D environment, due to perspective projection, the same change in the 3-D environment produces a larger change in the foreground than in the background of the 2-D image. Thus, it follows that people might be more sensitive to changes in the foreground than in the background.

We also found a small effect of confidence, such that more confident responses were slightly more likely to be associated with accurate responses than were less confident responses. Finally, females were slightly more likely to correctly identify inconsistent reflection scenes than males were.^10^ The results of the GEE analysis for the consistent scenes revealed that none of the variables had an effect on subjects’ ability to accurately identify consistent reflection scenes.

In Experiment 4, subjects correctly classified a mean 50% of the scenes indicating their performance was no better than expected by chance alone. These results suggest that people have an extremely limited ability to identify when the reflections within a scene are consistent versus inconsistent; this is despite the fact that each scene contained sufficient information to determine the answer objectively. There are at least two reasons, however, for why it might have been too difficult to make use of the reflection vanishing points in Experiment 4. First, the scenes’ reflection vanishing point was located a mean 1,386 pixels from the image center (image resolution 960 × 720 pixels), a distance that might have been sufficiently large to make it difficult to use this information. Second, in the inconsistent scenes, the mean angle difference between the target object’s reflection vanishing point and the reflection vanishing point for the rest of the scene was just 1°. Perhaps these two factors made it too difficult for subjects to use the geometrical information and locate the reflection vanishing point. For these reasons, and because relatively little research has examined people’s perception of reflections, we conducted a further experiment with new stimuli. For the new stimuli we decreased the distance of the reflection vanishing point from the center of the image and increased the angle difference from the scene reflection vanishing point to the vanishing point for the target object and its inconsistent reflection.

### Experiment 5

#### Method

##### Subjects and design

A total of 97 subjects (*M* = 25.5 years, *SD* = 9.0, range: 15–57 years, 58 men, 36 women, three chose not to disclose their gender) completed the study online. Eight additional subjects were excluded from the analyses because they experienced technical difficulties. The design was identical to that of Experiment 4.

##### Stimuli

**Fig. 8:**
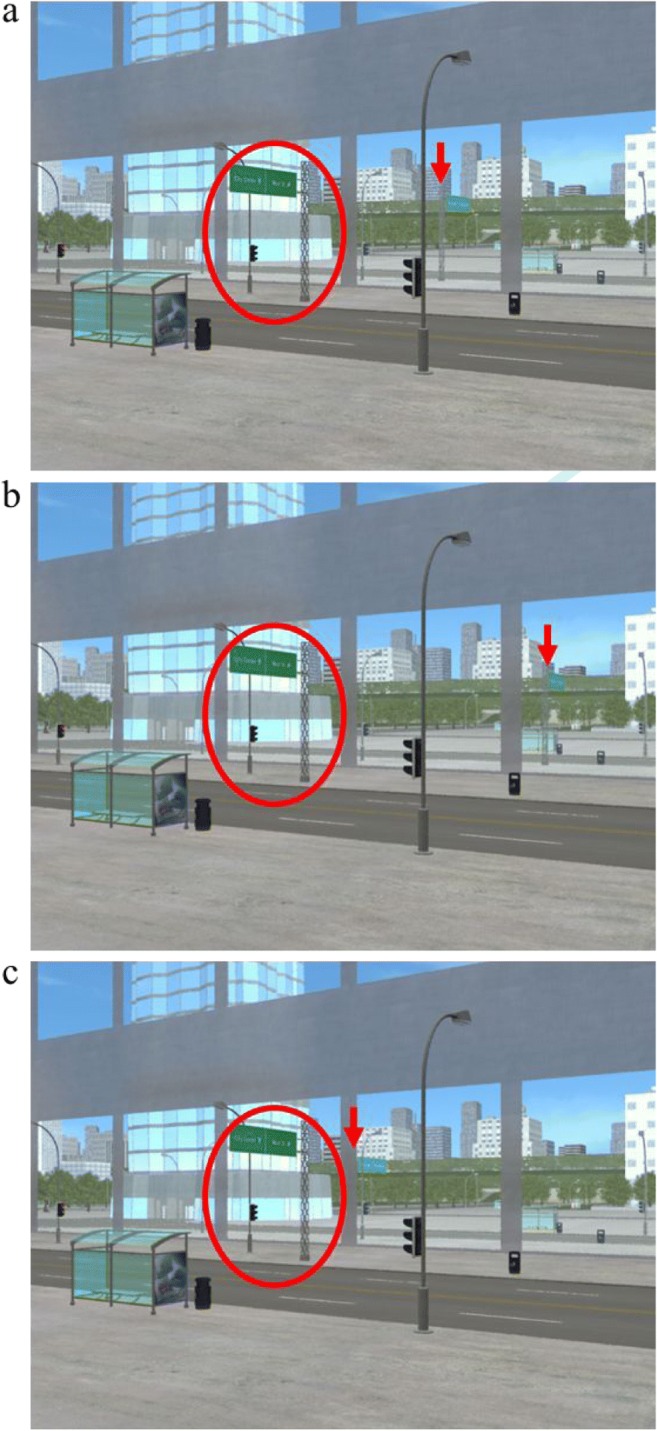
Example of the consistent and two inconsistent versions of a scene. **a** Original image with consistent reflections. **b** Forward inconsistent reflection image, with the target street sign reflection moved forward, of the consistent position. **c** Backward inconsistent reflection image, with the target street sign reflection moved backward, of the consistent position. Each subject saw this city scene just once, and they were randomly shown **a**, **b**, or **c**. In the example images the target street sign is shown in a red circle, and a red arrow indicates the location of the street sign’s reflection. (Color figure online)

We created the stimuli following the procedure in Experiment 4, with two exceptions. First, we changed the viewing perspective to bring the reflection vanishing point closer to the center of the image—the mean distance was 660 pixels (cf. Experiment 4: 1,386 pixels). Second, to increase the angle difference between the original, consistent reflection and the manipulated, inconsistent reflection, we moved the street sign 10 m on the *z* axis (cf. Experiment 4: 7 m). The resulting mean angle difference between the reflection vanishing point for the inconsistent reflections and the reflection vanishing point for the rest of the scene was 3.5° (cf. Experiment 4: 1°; see Fig. 8 for an example of the consistent and inconsistent versions of a scene).

##### Procedure

The procedure was identical to that used in Experiment 4.

#### Results and discussion

##### Overall accuracy

Overall, subjects correctly classified a mean 73% of the reflection scenes (cf. Experiment 4: 50%). Subjects showed a reasonably good ability to discriminate between consistent (72% correct) and inconsistent (75% correct) reflection scenes, *d'* = 0.91, 95% CI [0.72, 1.10]. Unlike in Experiment 4, these results suggest that subjects have some ability to identify consistent and inconsistent reflections. Perhaps, then, subjects make use of the reflection vanishing point to objectively judge the consistency of the reflections in a scene, but only in instances where the vanishing point is relatively easy to determine. In addition, in contrast to Experiment 4, subjects did not show a bias toward saying that reflections were inconsistent, *c* = −0.03, 95% CI [−0.12, 0.07]. This finding offers some support for our suggestion that people might only rely on perceptual biases to make judgements about reflections when there is a lack of information available to make a more informed decision.

##### Individual factors and image metrics

As in Experiment 4, we conducted two exploratory GEE analyses—one for the inconsistent and one for the consistent reflection scenes. Preliminary analyses revealed a variance inflation factor of 11.8 for the angle difference variable, suggesting that this variable was correlated with one or more of the other predictor variables, therefore we removed the angle difference variable from the analyses. The results of the GEE analyses are shown in Table 4. Replicating our finding from Experiment 4, more confident responses were slightly more likely to be associated with accurate responses than less confident responses were. There was also an effect of distance from the center of the image to the reflection vanishing point; scenes in which the reflection vanishing point was closer to the image were more likely to be identified as inconsistent compared with scenes in which the reflection vanishing point was further from the image. This time, however, we did not find an effect of reflection position or gender.

**Table 4:** Experiment 5. Results of the GEE binary logistic models to determine variables that predict accuracy in the reflection task

Predictor	Inconsistent		Consistent			
	*B*	*OR* [95% CI]	*p*	*B*	*OR* [95% CI]	*p*
Reflection position = forward	0.04	1.05 [0.53, 2.08]	.90	–	–	–
Confidence	0.02	1.02 [1.01, 1.04]	.002	−0.01	0.99 [0.97, 1.01]	.35
Gender = female	−0.40	0.67 [0.29, 1.57]	.36	−0.43	0.65 [0.28, 1.53]	.33
Vanishing point distance	−0.17	0.84 [0.71, 1.00]	.04	−0.21	0.81 [0.71, 0.93]	.002
Interest in photography = interested	0.08	1.08 [0.47, 2.48]	.85	−0.12	0.89 [0.42, 1.90]	.76
Video gaming = frequent (at least twice a month)	−0.35	0.70 [0.30, 1.64]	.42	0.77	2.16 [0.98, 4.76]	.06
Response time	0.00	1.00 [0.98, 1.02]	.88	0.00	1.00 [0.99, 1.01]	.89

*Note.* CI = confidence interval. *B* and odds ratios (*OR*) estimate the degree of change in accuracy associated with one unit change in the independent variable. An odds ratio of 1 indicates no effect of the independent variable on accuracy; values of 1.5, 2.5, and 4.0 are generally considered to reflect small, medium, and large effect sizes, respectively (Rosenthal, 1996). The category order for factors was set to descending to make the reference level zero. The reference groups are reflection position = back, video-game playing = infrequent (never/less than once a month/about once a month), gender = male, interest in photography = not interested. Response time, confidence, and reflection vanishing point distance were added as continuous variables. The three subjects who chose not to disclose their gender were excluded from these analyses, leaving a total sample of *n* = 94. The reflection position predictor variable was not applicable in the consistent reflection scenes.

Considering the consistent reflection scenes, the GEE analysis revealed that only one variable had an effect on accuracy—the distance of the reflection vanishing point. As with the inconsistent scenes, when the reflection vanishing point was closer to the center of the image the scenes were more likely to be identified as consistent compared with when it was further from the center. It appears, then, that people might be able to make use of the geometrical information provided in the scenes to objectively judge the validity of the reflections when the reflection vanishing point is closer to the center of the image.

That said, another possibility is that moving the inconsistent reflections further from the consistent position in Experiment 5 than in Experiment 4 made it more visually apparent when the reflections were consistent versus inconsistent. If so, perhaps even based on a visual inspection of the scene, the correspondence between the object and its reflection did not match people’s subjective expectation of how it should look—including for the backward inconsistent reflections.

So why did subjects perform better on the reflection task in Experiment 5 than in Experiment 4? Given that we made two changes to the stimuli between Experiments 4 and 5, there are two possible reasons. One possibility is that creating scenes with the reflection vanishing point closer to the center of the image made it easier for people to use geometric analysis to work out the answer. A second possibility is that the bigger physical distance between the consistent and inconsistent reflection position made it easier to make a subjective judgement about the consistency or inconsistency of the reflections in the scene. To check which explanation best accounts for people’s better performance in Experiment 5, we ran a further experiment in which we changed only one variable. In Experiment 6, the scene reflection vanishing point remained the same as in Experiment 5, but we decreased the distance between the inconsistent and the consistent reflection position to match the distance in Experiment 4.

### Experiment 6

#### Method

##### Subjects and design

A total of 120 subjects (*M* = 30.9 years, *SD* = 13.7, range: 14–77 years, 53 men, 62 women, five chose not to disclose their gender) completed the study online. A further 10 subjects were excluded from the analyses because they experienced technical difficulties. The design was identical to that of Experiments 4 and 5.

##### Stimuli

The stimuli remained the same as in Experiment 5 with one exception: We decreased the distance that we moved the street sign reflection from its consistent position when creating the inconsistent scenes. In Experiment 5, we moved the street sign 10 m on the *z* axis relative to the original street sign position; this time we moved it the same distance as in Experiment 4 (7 m). The resulting mean angle difference between the reflection vanishing point for the inconsistent reflections and the reflection vanishing point for the rest of the scene was 2.4° (cf. Experiment 5: 3.5°).

##### Procedure

The procedure was identical to that used in Experiments 4 and 5.

#### Results and discussion

##### Overall accuracy

Overall, subjects correctly classified a mean 62% of the reflection scenes (cf. Experiment 4: 50%; Experiment 5: 73%). Subjects showed a fairly limited ability to discriminate between consistent (68% correct) and inconsistent (55% correct) reflection scenes, *d'* = 0.46, 95% CI [0.27, 0.65]. Our results show that subjects in Experiment 6 correctly classified a mean 24% more of the reflection scenes as consistent or inconsistent than subjects in Experiment 4, a difference of 12 percentage points. This difference in performance suggests that the position of the reflection vanishing point might influence people’s ability to distinguish between consistent and inconsistent reflection scenes. Yet subjects in Experiment 6 correctly classified a mean 15% fewer of the scenes as consistent or inconsistent than subjects in Experiment 5, a difference of 11 percentage points, indicating that the extent of the inconsistency might also have an effect on people’s performance. In line with this suggestion, in Experiment 5 the inconsistent reflections were positioned further from the consistent position than in Experiment 6, and we did not find evidence of a response bias. Yet in Experiment 6, subjects showed a bias toward accepting the reflection scenes as consistent, *c* = 0.12, 95% CI [0.04, 0.21]. Taken together, these results suggest that people might have a relatively conservative criterion for judging that the reflections in a scene are inconsistent. As such, it is possible that people have a perceptual threshold for detecting reflection inconsistencies—that is, there is a point at which the inconsistent reflections are close enough to the consistent position that people will find it extremely difficult to detect the inconsistency; instead, they simply accept the reflection as consistent.

##### Image metrics and individual factors

Table 5 shows that none of the variables had a significant effect on subjects’ ability to accurately identify consistent reflection scenes. Only one variable had an effect on subjects’ ability to accurately identify inconsistent reflection scenes: Replicating Experiment 4, we found an effect of reflection position. Scenes in which the reflection was moved forward of its consistent position were more likely to be identified as inconsistent than scenes in which the reflection was moved backward. This result might simply be an effect of perspective projection: The same change in the 3-D environment produces a larger change in the foreground than in the background of the 2-D image, and thus people are more sensitive to changes in the foreground than in the background.

**Table 5:** Experiment 6. Results of the GEE binary logistic models to determine variables that predict accuracy in the reflection task

Predictor	Inconsistent			Consistent		
	*B*	*OR [95% CI]*	*p*	*B*	*OR [95% CI]*	*p*
Reflection position = forward	1.13	3.10 [1.84, 5.23]	<.001	–	–	–
Confidence	0.01	1.01 [0.99, 1.02]	.42	0.01	1.01 [1.00, 1.03]	.12
Gender = female	0.21	1.23 [0.65, 2.31]	.52	−0.34	0.71 [0.32, 1.57]	.40
Vanishing point distance	−0.09	0.91 [0.81, 1.03]	.13	−0.08	0.93 [0.81, 1.06]	.28
Interest in photography = interested	−0.03	0.97 [0.52, 1.79]	.92	0.46	1.59 [0.82, 3.10]	.17
Video gaming = frequent (at least twice a month)	0.36	1.43 [0.75, 2.73]	.28	0.39	1.47 [0.71, 3.06]	.30
Response time	0.00	1.00 [0.99, 1.01]	.59	−0.01	0.99 [0.98, 1.00]	.15

*Note.* CI = confidence interval. *B* and odds ratios (*OR*) estimate the degree of change in accuracy associated with one unit change in the independent variable. An odds ratio of 1 indicates no effect of the independent variable on accuracy; values of 1.5, 2.5, and 4.0 are generally considered to reflect small, medium, and large effect sizes, respectively (Rosenthal, 1996). The category order for factors was set to descending to make the reference level zero. The reference groups are reflection position = back; video-game playing = infrequent (never/less than once a month/about once a month); gender = male; interest in photography = not interested. Response time, confidence, and reflection vanishing point distance were added as continuous variables. The five subjects who chose not to disclose their gender were excluded from these analyses, leaving a total sample of *n* = 115. The reflection position predictor variable was not applicable in the consistent reflection scenes.

Our finding that there was an influence of reflection position in Experiments 4 and 6, but not in Experiment 5, suggests a perceptual threshold for detecting when reflections in a scene are inconsistent. Put simply, in Experiment 5, when the inconsistent reflections were moved 10 m from the consistent position, we did not find a reliable effect of reflection position on subjects’ ability to identify the inconsistent scenes. Yet in Experiments 4 and 6 when the inconsistent reflections were moved a smaller distance (7 m) from the consistent position, we did find a reliable effect of reflection position on performance. These findings suggest that there might be a point at which the inconsistent reflection becomes different enough from its consistent position to make the inconsistency noticeable.

That said, it is important to note that adjusting the distance of the inconsistent reflections from the original position also changes the angle difference between the scene reflection vanishing point and the reflection vanishing point for the inconsistent reflection—as the distance increases, so does the angle difference. Thus, we are not able to isolate the two factors and test them individually. Although our results appear to support the notion of a perceptual threshold in people’s ability to subjectively determine the validity of the reflections based on a visual inspection of the scene, we cannot rule out an alternative explanation. Instead, it remains possible that changes to the angle difference between the scene reflection vanishing point, and the reflection vanishing point for the inconsistent reflection affects people’s ability to use the geometric information in the scene.

To establish whether the angular difference or the vanishing point distance was more strongly associated with accuracy on the reflection task, we combined the data from Experiments 4, 5, and 6 (*N* = 296) and calculated the mean angular difference and the mean vanishing point distance. For each subject, we calculated the number of correct responses on the inconsistent trials—0, 1, or 2.

We ran an ordinal logistic regression with two independent variables: (1) the mean angular difference from the scene’s reflection vanishing point to the reflection vanishing point of the target object and its inconsistent reflection, and (2) the mean distance from the center of the image to the reflection vanishing point. Our results revealed that both of these variables had an effect on the likelihood of responding correctly. First, an increase in the angular difference was associated with an increase in the number of correct responses (*OR* = 2.14, 95% CI [1.53, 3.00]). Second, although only a small effect, an increase in distance to the vanishing point was associated with an increase in the number of correct responses (*OR* = 1.05, 95% CI [1.01, 1.08]). At first glance this effect of vanishing point distance seems somewhat surprising—intuitively, a vanishing point closer to the center of the image would be easier to determine than one that is located further away. Therefore, perhaps subjects did not make use of the reflection vanishing point when deciding whether the reflections in each scene were consistent or inconsistent. An accurate reconstruction of the reflection vanishing point offers an objective technique for verifying the (in)consistency of the reflections in a scene. Nonetheless, this reconstruction is a perceptually challenging task that requires accurately locating numerous object and corresponding reflection points as well as mapping these corresponding points as lines that extend beyond the image plane. Accordingly, our findings suggest that when deciding whether the reflections in a scene were consistent or inconsistent, subjects tended to rely on the appearance of the in-plane image cues rather than attempting to determine the reflection vanishing point.

In summary, across three experiments we examined people’s ability to identify whether scenes contained consistent or inconsistent reflections. The results of Experiment 4 suggest that people have an extremely limited ability to identify when reflections in a scene are consistent or inconsistent. Yet, in Experiment 5, when we brought the reflection vanishing point closer to the center of the image and also moved the inconsistent reflections further from the consistent position, subjects’ performance on the task improved. Moreover, in Experiment 6 we kept the vanishing point position the same as in Experiment 5, but decreased the distance between the inconsistent and the consistent reflection position to match the distance in Experiment 4. Subjects then correctly classified fewer of the consistent and inconsistent scenes than in Experiment 5, but more than in Experiment 4. Thus, it seems that people’s understanding of how reflections should appear in images is not straightforward, but rather might depend on various factors, including the location of the reflection vanishing point and the extent of the inconsistency.

## General discussion

Across seven experiments, we found that people had a limited ability to determine whether the shadows or reflections within a scene were consistent or inconsistent, and this ability depended to some degree on the size of the inconsistency. Given the ubiquity of image manipulation, it is important to consider what our results reveal about how people process visual information. In doing so, we aim to highlight other possible avenues for improving people’s ability to distinguish between real and fake images.

The current findings add to our theoretical understanding of how the visual system processes information. Although people seemingly experience a detailed and coherent picture of the world, the striking finding that people are slow to detect even large changes that occur during a real or simulated eye blink suggests that this is not the case (e.g., Pashler, 1988; Simons, 1996; Simons & Levin, 1997). Why, then, do people have the impression of observing a richly detailed and coherent world? From the standpoint of coherence theory, this impression is the result of a visual system that generates a sparse and incomplete representation of the scene whereby most parts are represented only at a preattentive level (Rensink, 2000, 2002). This incomplete representation is achieved via a low-level subsystem that involves an automatic and continual processing of the visual scene to generate simple visual elements without the awareness of the observer. According to coherence theory, a limited-capacity attentional subsystem can form a subset of visual elements into a coherent and detailed object representation—this is the basis of conscious perception. Owing to a finite attentional capacity, these detailed, conscious representations are only created for the objects needed for the task at hand. The attentional subsystem is guided via a combination of low-level factors (e.g., salience of individual elements) and high-level factors (e.g., knowledge) to create representations of the appropriate objects at the appropriate time. Together, these subsystems can provide the impression that perceptions are stable and highly detailed, even though a complete representation of the scene is never constructed.

Furthermore, previous research has shown that when people take an effortful approach to attend to the details of a scene, aspects such as shadows and reflections rarely receive attention (Ehinger et al., 2016; Rensink, 2002; Rensink & Cavanagh, 2004; Sareen, Ehinger, & Wolfe, 2015). As such, researchers have suggested that shadow and reflection information is typically processed by the low-level visual system that rapidly identifies and then discounts these features (Rensink & Cavanagh, 2004; Sareen et al., 2015). Indeed, this insensitivity to shadow and reflection information can account for our finding that, even when cued to the target object and its shadow, people struggled to make an accurate subjective judgement about whether or not these aspects of the scene were consistent or inconsistent. Specifically, if people discard information about shadows and reflections at an early stage of visual processing, then it follows that they will not have an opportunity to learn how these aspects should appear. Yet when we looked more closely at the results of our shadow and reflection experiments, we found that the extent of the inconsistency influenced people’s performance. That is, under the current experimental conditions, people were more likely to detect inconsistent shadows and reflections when they were positioned further from the correct position. As such, our results fit with the notion of a perceptual threshold for detecting lighting inconsistencies based on cues within the image plane (Lopez-Moreno et al., 2010; Tan et al., 2015).

It seems possible, then, that there is a discernible point at which the inconsistent shadows are sufficiently different from the consistent position to be detected preattentively. But for shadow inconsistencies that do not pass this perceptual threshold, a more effortful strategy is required for people to detect the inconsistency. Indeed, our results from the three reflection experiments revealed a similar possibility—that people might have a perceptual threshold for noticing inconsistencies in the reflections in a scene. People’s ability to identify inconsistent reflections appeared to be dependent upon a number of factors. In particular, in Experiment 5, when the inconsistent reflections were moved further from the consistent position than in Experiments 4 or 6, people detected the inconsistencies 75% of the time (cf. 58% in Experiment 4 and 55% in Experiment 6). As such, even if forgers leave behind inconsistencies in the shadows or reflections in an image, our results suggest that people are unlikely to capitalize on such tell-tale signs to help to detect forgeries in the real world. Essentially, our results suggest that when image manipulations create quite large inconsistencies in the shadows or reflections of the scene, people might be able to detect the image as a fake. When the manipulations were smaller, but still quite “wrong,” people were much less likely to be able to detect which images had been altered. Yet it is important to note that subjects in our study were explicitly asked to examine the images and determine if the image had been manipulated. Moreover, they were even cued as to exactly which shadow or reflection might have been changed. Despite this, and the fact that they took a relatively long time examining the images (the shortest mean response time per image in an experiment was 16.9 seconds, and the longest was 57.3 seconds), the detection rates were still far from perfect, even with the larger changes. In more casual and limited viewing conditions, the tolerances for accepting faked images as real with shadow and reflection inconsistencies is likely to be much higher than those reported here.

We specifically explored people’s ability to identify inconsistencies when there was enough information in the scene to use two types of geometric analyses based on shadow and reflection information in the scene. These types of analyses form the basis of some of the digital image forensic computer programs that can help to verify the authenticity of images (e.g., Kee et al., 2013; O’Brien & Farid, 2012). Yet our results suggest that people are reasonably insensitive to inconsistencies in shadows and reflections. This finding is troubling when considering people’s ability to use these details to detect visual misinformation; it does, however, also suggest that forgers might make mistakes and leave behind clues in the visual details when editing images. This result suggests that (i) the visual system does not contain machinery that automatically implements the proposed geometric analysis strategies for reconstructing light source or vanishing point locations and signals the results to higher levels of processing, and (ii) people do not spontaneously apply such strategies in an intentional goal-directed manner, even when there is sufficient information in an image to allow them to be applied.

The rise of photo manipulation paired with people’s apparent insensitivity to such manipulation has consequences across myriad domains, from law enforcement (when photos are used as evidence in court) through to politics (when photos are used to promote a political agenda). Moreover, research shows that doctored photos (and videos) can influence people’s beliefs, memories, intentions and behaviors (e.g., Nash, 2017; Sacchi, Agnoli, & Loftus, 2007; Wade, Garry, Read, & Lindsay, 2002; Wade, Green, & Nash, 2010). The current research does not provide a solution to the complex problem of how to detect image forgeries, but the results suggest that people might frequently neglect potentially useful cues in images. A priority for future research should be to explore whether, with training, people can learn to use these shadow-based and reflection-based analyses. Encouragingly, researchers have shown that performance on many types of perceptual tasks can improve as a result of training (e.g., Ellison & Walsh, 1998; Porter, Tales, & Leonards, 2010; Sireteanu & Rettenbach, 1995, 2000). That said, these shadow-based and reflection-based analyses rely on the visibility of a number of distinct features to allow one to determine the corresponding points on the object and the object’s shadow/reflection—when these features are not distinct (for example, with “soft shadows”), then this task will still be very challenging. What’s more, given that successful use of the proposed shadow-based and reflection-based analyses requires a precise localization of features, it is unlikely to be something that the human visual system implements automatically. Rather, using these techniques when viewing scenes in the real world will require an effortful and careful mapping of object-shadow/reflection features—a task that is already known to be difficult (e.g., Dee & Santos, 2011; Mamassian, 2004).

The seven experiments reported are an initial exploration of people’s ability to use errors in the physical properties of images to help them to detect forgeries. As such, we used computer-generated stimuli that afforded us great precision and the ability to carefully control the scenes and manipulations. Our computer-generated stimuli consisted of complex, photorealistic scenes. But, of course, the use of such scenes might limit how far the results can be generalized. Moreover, our results are in line with the previous literature showing people’s limited ability to identify inconsistencies in real-world scenes (Kasra et al., 2016; Nightingale et al., 2017). Therefore, it seems likely that the findings reported here will apply to other more natural types of stimuli and an important next step is to test this suggestion empirically.

In sum, our findings suggest that people might frequently neglect information that could help them to accurately determine whether images are real or fake. It is clear that image manipulation is not going away; as digital technology improves, forgeries are only going to become more visually compelling, and thus more difficult to detect. The challenge now is to try to find ways to prevent people from being fooled by manipulated photos.

## Figures and Tables

**Fig. 9:**
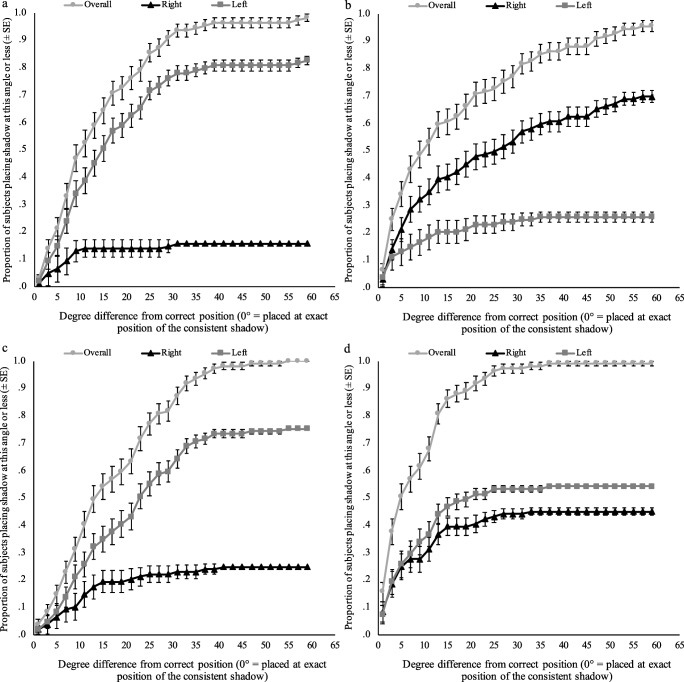
Cumulative proportion of responses made by each angle difference level for (**a**) Scene 1, (**b**) Scene 2, (**c**) Scene 3, and (**d**) Scene 4. The light-gray line with circle markers shows the overall proportion of responses made by each angle difference level and therefore includes both negative and positive values—for example, 10° on the graph represents responses between −10° and +10°. We also calculated the number of positive and negative responses made by each angle difference level and displayed these as a proportion of the total responses. The black line with triangle markers shows the proportion of negative angle difference responses (shadow to the right of the consistent position). The dark-gray line with square markers shows the proportion of positive angle difference responses (shadow to the left of the consistent position). Error bars represent standard error of the mean.

**Fig. 10:**
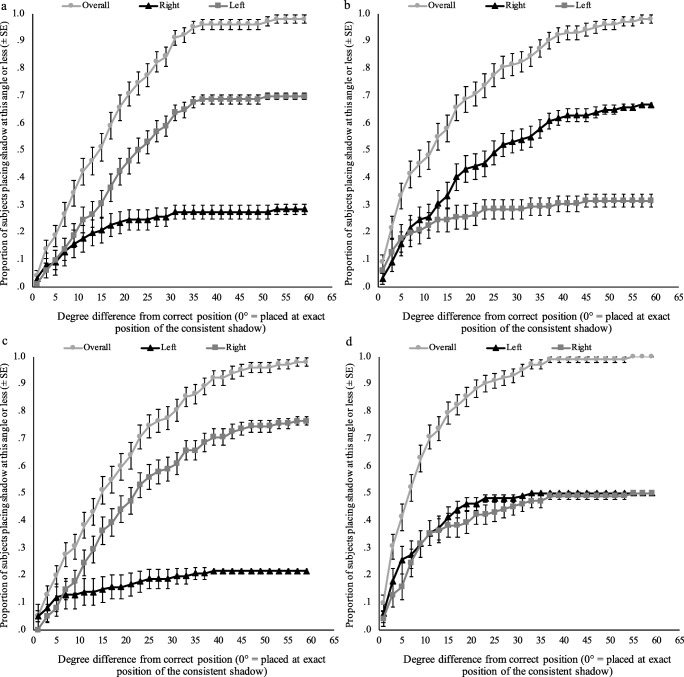
Cumulative proportion of responses made by each angle difference level for (**a**) Scene 1, (**b**) Scene 2, (**c**) Scene 3, and (**d**) Scene 4. The light-gray line with circle markers shows the overall proportion of responses made by each angle difference level and therefore includes both negative and positive values—for example, 10° on the graph represents responses between −10° and +10°. We also calculated the number of positive and negative responses made by each angle difference level and displayed these as a proportion of the total responses. The black line with triangle markers shows the proportion of negative angle difference responses (shadow to the right of the consistent position). The dark-gray line with square markers shows the proportion of positive angle difference responses (shadow to the left of the consistent position). Error bars represent standard error of the mean.

## Appendix A

**Table 6:** List of questions from Experiments 1, 4, 5, and 6

Question	Response options								
Please enter your age in the box (If you would prefer not to say, please write n/a)									
What is your gender?	Male	Female	Prefer not to say						
Do you have a personal or professional interest in photography?	Yes	No							
Generally, would you describe yourself as more of a science or arts person?	Science	Arts							
How often do you play video games?	Daily	5-6 times a week	3-4 times a week	Once or twice a week	A couple of times a month	About once a month	Less than once a month	Never	
How long is your typical playing session?	Less than an hour	1-2 hours	2-4 hours	4-6 hours	6-8 hours	8-10 hours	10-12 hours	12 hours or more	
Approximately how old were you when you played your first video game?	Before the age of 6	Aged 6-9	Aged 10-13	Aged 14-17	18+				
Approximately how many different video games in any format have you played to date?	1-5	6-20	21-50	51-100	Over 100				
And what type of video games do you play? Please select all that apply.	Role-playing	Adventure	Sports	Puzzle/card/board	Simulation	Strategy	Action	Rhythm (music games)	Driving

*Note.* Only age and gender questions asked in Experiments 2a, 2b, and 3

**Table 7:** Mean and median response time per image in each experiment

Experiment	Response time per image in seconds	
	*M* (*SD*)	*Mdn* (interquartile range)
1	16.9 (9.1)	14.1 (10.2, 23.1)
2a	57.3 (419.3)	23.9 (15.8, 39.7)
2b	34.7 (27.3)	28.6 (19.1, 42.5)
3	26.3 (50.2)	19.4 (12.3, 29.6)
4	23.9 (24.8)	17.4 (9.9, 25.5)
5	23.0 (15.9)	18.2 (13.5, 27.9)
6	45.0 (144.1)	26.2 (18.8, 38.6)

## Appendix B

### Experiment 2a

#### Method

##### Subjects and design

A total of 109 subjects (*M* = 25.1 years, *SD* = 9.7, range: 14–64 years, 41 women, 65 men, three chose not to disclose their gender) completed the task online. Two additional subjects were excluded from the analyses because they experienced technical difficulties. We used a within-subjects design.

##### Stimuli

We used the same five original city scenes as in Experiment 1. We removed the target lamppost’s shadow from each of the original scenes and saved the shadows as new PNG image files. We developed a program in HTML to display the original scene with the shadow image overlaid but rotated at a random angle between −45° and +45° of the correct shadow location. As such, there was one version of each scene, but the initial position for the target shadow was randomized.

##### Procedure

Subjects were told that they could assume that “each of the scenes is illuminated by a single light source, such as the sun.” Subjects were given a practice trial before being presented with the four city scenes in a random order. To cue the target lamppost, the scene first appeared with a red ellipse around the lamppost, which disappeared after 2 s. For each scene, subjects were instructed to use the left and right arrow keys to change the shadow rotation and press enter when they believed the shadow was consistent with the other shadows in the scene. Subjects could rotate the shadow ±180° in increments of 0.1° from the correct position which was designated 0°. Clockwise rotation gave positive values from 0.1° to 179.9°, indicating that the shadow was placed left of its consistent position. Counterclockwise rotation gave negative values from −0.1° to −179.9°, indicating that the shadow was placed right of its consistent position. Subjects were given unlimited time to respond and were then asked to rate their confidence in their decision using a 100-point Likert-type scale from 0 (*not at all confident*) to 100 (*extremely confident*). Finally, subjects were asked whether they had experienced any technical difficulties while completing the experiment.

#### Results and discussion

##### Overall accuracy

The range of shadow positions makes it possible to measure accuracy in several ways. Taking an extremely conservative approach, just 7% of the shadows were positioned between −1° and +1°, across the four scenes, 95% CI [5%, 10%]. Taking a more lenient approach, 51% of the shadows were positioned between −10° and +10°, 95% CI [46%, 56%]. And 95% of the shadows were positioned between −40° and +40° of the correct location, 95% CI [93%, 97%]. The remaining 5% of shadows were positioned as widely as −150° and +150° of the correct location. As in Experiment 1, these results indicate that subjects had only a basic understanding about where an object’s shadow must cast to be consistent with the light source. Our results also offer empirical support for the notion of a perceptual threshold for noticing lighting inconsistencies in images (Lopez-Moreno et al., 2010; Tan et al., 2015). To more precisely estimate the perceptual system’s sensitivity to lighting inconsistencies than in previous research, we allowed subjects to rotate the target shadow through 360° at 0.1° increments. Despite this high level of control, subjects were still willing to rotate the target shadow to a relatively wide range of positions that were inconsistent with the scene lighting. Therefore, people’s perceptual threshold for accepting shadows as consistent with a single light source could be surprisingly wide.

To further understand subjects’ perception of illumination inconsistencies, we calculated accuracy by scene. Fig. 9 shows the angle difference from the correct shadow position (0°) on the horizontal axis where smaller angle differences indicate that the shadow was positioned closer to its correct position than larger angle differences. The cumulative proportion of responses that were made by each angle difference level are presented on the vertical axis. The results indicate that subjects’ accuracy on the shadow task varied by scene. Subjects were most accurate in Scene 4, where 50% correctly positioned the target shadow between −5° and +5° of the correct location, 95% CI [41%, 60%]. Subjects were least accurate in Scene 3, where only 15% positioned the target shadow between −5° and +5° of the correct location, 95% CI [8%, 21%]. Consistent with this finding, we know that various factors affect the detection of illumination inconsistencies, including the scene content and layout (Tan et al., 2015; Xia, Pont, & Heynderickx, 2016). Specifically, if a scene contains numerous objects that are all orientated in the same direction, then lighting inconsistencies are typically easier to notice (Enns & Rensink, 1990). Therefore, our results support the idea that the perception of shadow inconsistencies is influenced by the configuration of the scene (Ostrovsky et al., 2005; Tan et al., 2015; Xia et al., 2016).

#### Preference for shadows to the left or right

To determine whether subjects showed a preference to position the target shadow to the left or to the right of the true position, we classified each response as left or right of the correct position. A mean 20% more shadows were positioned to the left than to the right of the correct location, *M*_diff_ 95% CI [12%, 28%]. Yet this trend differed across the four scenes (see Fig. 9). Subjects were more likely to rotate the shadow to the left in Scenes 1 and 3 (Scene 1: 84%, 95% CI [77%, 91%]; Scene 3: 75%, 95% CI [67%, 83%]), but to the right in Scene 2 (Scene 2: 73%, 95% CI [65%, 82%]). A similar proportion of subjects positioned the shadow to the left (54%) as to the right (46%) in Scene 4.

In sum, the results from Experiment 2a further support the idea that subjects typically make imprecise judgements about where shadows must be positioned to be consistent with a single light source. That said, because the rotatable shadow in Experiment 2a was taken from the consistent version of the scene, it is possible that the shape of the shadow provided subjects with an additional cue to its correct position. Therefore these results might actually *overestimate* people’s ability to position a target shadow so it is consistent with the scene light source. To check this possibility, in Experiment 2b subjects could both rotate the shadow and change the size of the shadow.

### Experiment 2b

#### Method

##### Subjects and design

A total of 102 subjects (*M* = 25.1 years, *SD* = 10.3, range: 14–59 years, 47 women, 50 men, five chose not to disclose their gender) completed the task online. Four additional subjects were removed because they experienced technical difficulties. The design was identical to that of Experiment 2a.

##### Stimuli and procedure

We used the same stimuli and program as in Experiment 2a, but with one modification—subjects could adjust both the size and the orientation of the shadow. The size was randomized so that the target shadow initially appeared between 0.5 and 1.5 times its correct scale isotropically. Subjects were instructed to use the left and right arrow keys to change the shadow rotation and the up and down arrow keys to change the shadow size, and to press enter when they believe the shadow was consistent with the other shadows in the scene.

#### Results and discussion

##### Overall accuracy

Replicating the result from Experiment 2a, across the four scenes, just 7% of the shadows were positioned between ±1°, 95% CI [4%, 9%]. With the more lenient classification for accuracy on the task, 46% of shadows were positioned between ±10° (cf. Experiment 2a: 51%), 95% CI [41%, 51%] and 95% were positioned between ±40° of the correct location, 95% CI [93%, 97%]. Of the remaining shadows, 4.8% were positioned as widely as −124° and +124° of the correct location (one outstanding shadow was positioned at −170°). Recall that the results from Experiment 2a showed that subjects’ accuracy on the shadow task varied by scene; as Fig. 10 illustrates, this finding replicated in Experiment 2b. Again, subjects were more accurate in positioning the shadow in Scene 4 than in the other three scenes. In Scene 4, 50% of shadows were positioned between ±6.7° of the correct location, 95% CI [40%, 60%]. Also replicating Experiment 2a, subjects were least accurate in Scenes 1 and 3, where the target shadow was positioned between ±6.7° of the correct location by only 25% and 26% of subjects, respectively (Scene 1: 95% CI [17%, 34%]; Scene 3: 95% CI [18%, 35%]).

##### Preference for shadows to the left or right

Replicating Experiment 2a, collapsed across the four scenes, subjects positioned 16% more of the shadows to the left of the correct position than to the right, *M*_diff_ 95% CI [7%, 25%]. Moreover, this directional preference was context dependent—it varied by scene. As Fig. 10 shows, subjects were more likely to move the shadow to the left in Scenes 1 and 3, but more likely to move them to the right in Scene 2. In Scene 4, subjects were equally likely to have positioned the shadow to the left or right of its correct location.

##### Shadow size

For the size of shadow analysis, the error is qualified as the ratio of (reported size)/(true size). As such, a value of 1 represents the correct size for the shadow, values greater than 1 indicate the shadow was adjusted to be larger than its correct size, while values smaller than 1 indicate that the shadow was adjusted to be smaller. Overall, subjects adjusted the shadows to appear slightly larger than the shadows’ correct size, *M*_diff_ = 0.03, 95% CI [0.01, 0.06]. Therefore, subjects’ accuracy on this aspect of the task was reasonably good.

## Footnotes

1 In a 3-D scene, a shadow point, object point, and the light source lie on a straight line. Assuming a rectilinear projection, the transformation of the 3-D world coordinates to 2-D image coordinates means that in a 2-D image of the scene, the line connects the images of the shadow point and object point, and intersects the projected image of the light source (e.g., Kee et al., 2013).

2 This analysis assumes that all of the shadows in the scene are cast by a single light source, which is reasonable to assume in daylight outdoor scenes, such as those in Fig. 1, but often cannot be assumed in indoor scenes.

3 A 95% level of assurance indicates that with 81 subjects we will achieve a margin of error no longer than the target of 0.25 on 95% of occasions (Cumming, 2014).

4 The rendering parameters for each scene and the link to the 3-D cityscape model are available upon request.

5 We used an extremely distant point light that for practical purposes acts like a directional light source. We also note that the geometric constraint for determining shadow consistency applies for both a local and distant light source.

6 The horizontal and vertical axes are defined as the image plane, and the *z* axis is the optical axis of the camera. To give a sense of scale, the width of the road in the scene is 14.3 m, and the lamppost height is 11.2 m.

7 Signal-detection measures *d'* and *c* were calculated at the participant level.

8 Although there are two types of reflection, in this paper we focus on specular reflection—the reflection of light from a smooth surface, such as a flat mirror or window.

9 Data cleaning resulted in the removal of five subjects, leaving the final sample at 79. In terms of the precision-for-planning analysis, this sample size provides a 93% level of assurance that we will achieve a margin of error no longer than the target of 0.25 (Cumming, 2014).

10 Given the plausibly small effect size, and also because we did not replicate the effect of gender in Experiments 5 or 6, it is possible that this finding occurred by chance.
